# Mesoporous MnCeO_*x*_ solid solutions for low temperature and selective oxidation of hydrocarbons

**DOI:** 10.1038/ncomms9446

**Published:** 2015-10-15

**Authors:** Pengfei Zhang, Hanfeng Lu, Ying Zhou, Li Zhang, Zili Wu, Shize Yang, Hongliang Shi, Qiulian Zhu, Yinfei Chen, Sheng Dai

**Affiliations:** 1 Chemical Sciences Division, Oak Ridge National Laboratory, Oak Ridge, Tennessee 37831, USA; 2 Institute of Catalytic Reaction Engineering, College of Chemical Engineering, Zhejiang University of Technology, Hangzhou 310014, China; 3 Materials Science and Technology Division, Oak Ridge National Laboratory, Oak Ridge, Tennessee 37831, USA; 4 Department of Chemistry, University of Tennessee, Knoxville, Tennessee 37996, USA

## Abstract

The development of noble-metal-free heterogeneous catalysts that can realize the aerobic oxidation of C–H bonds at low temperature is a profound challenge in the catalysis community. Here we report the synthesis of a mesoporous Mn_0.5_Ce_0.5_O_*x*_ solid solution that is highly active for the selective oxidation of hydrocarbons under mild conditions (100–120 °C). Notably, the catalytic performance achieved in the oxidation of cyclohexane to cyclohexanone/cyclohexanol (100 °C, conversion: 17.7%) is superior to those by the state-of-art commercial catalysts (140–160 °C, conversion: 3-5%). The high activity can be attributed to the formation of a Mn_0.5_Ce_0.5_O_*x*_ solid solution with an ultrahigh manganese doping concentration in the CeO_2_ cubic fluorite lattice, leading to maximum active surface oxygens for the activation of C–H bonds and highly reducible Mn^4+^ ions for the rapid migration of oxygen vacancies from the bulk to the surface.

## Abstract (web summary)

Precious metal free catalysts for aerobic oxidation of hydrocarbons are industrially useful materials. Here, the authors report a mesoporous maganese-cerium oxide solid solution that is highly active for the selective oxidation of C-H bonds in hydrocarbons under mild conditions.

## Graphical abstract

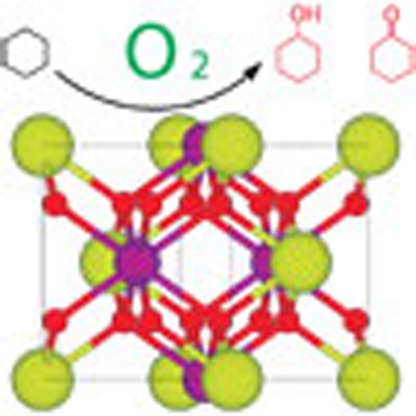

Aerobic oxidation has been considered as one of the most fundamental processes throughout organic synthesis and industrial chemistry^1^,^2^,^3^,^4^,^5^,^6^,^7^,^8^,^9^. Nowadays, realizing the selective oxidation of *sp*^3^ C–H bonds at low temperatures represents a critical challenge in the petroleum industry, because the current methods for the activation of C–H bonds generally require high temperature (for example, ∼600 °C for propane dehydrogenation) and excessive energy input, often resulting in uncontrolled product selectivity and undesirable cokes^10^,^11^,^12^,^13^,^14^,^15^,^16^,^17^,^18^,^19^,^20^. Among all C–H activation processes, the liquid-phase oxidation of cyclohexane to KA oil (K: cyclohexanone, A: cyclohexanol, production >2 × 10^6^ ton per year) is widely deployed in Nylon-6 and Nylon-6,6 production^21^. The industrial process proceeds with homogeneous Co/Mn carboxylate salts at 140–160 °C using 0.9–1.0 MPa air as an oxidant^3^. To minimize the overoxidation of KA oil to by-products, cyclohexane conversion is preferentially limited to <5%. Figure 1a summaries representative pathways to caprolactam (monomer for Nylon-6); the low cyclohexane conversion is definitely a bottleneck of the state-of-art technologies. This situation prompted catalysis scientists to explore the possibility of developing new catalysts, for example, *N*-hydroxyphthalimide^22^, metalloporphyrins^23^, transition metal ions-substituted molecular sieve catalysts^24^, supported gold catalysts^25^,^26^,^27^ and carbon-based catalysts^28^,^29^. However, several important issues are still unresolved, such as catalyst recycling and separation, the use of H_2_O_2_ or *tert*-butylhydroperoxide oxidants (the desired oxidant is air or O_2_) or dependence on noble metal elements. From the standpoint of chemical kinetics, the development of a heterogeneous catalyst that functions at lower temperature, may prevent deep radical oxidation to a large degree, ideally achieving a higher cyclohexane conversion.

Recently, MnO_*x*_–CeO_2_ hybrid catalysts with multiple redox states and high oxygen storage capacity have exhibited superior performance in several types of catalytic oxidation, such as ammonia oxidation, combustion of volatile organic compounds and CO oxidation^30^,^31^,^32^,^33^. Compared with either MnO_*x*_ or CeO_2_ (ceria), the significant decrease in reaction temperature enabled by the MnO_*x*_–CeO_2_ composite is very appealing, which directly evidences the synergistic interaction of MnO_*x*_ and CeO_2_ with more active oxygen species. These ‘reactive' oxygen species (for example, O_2_^−^, O_2_^2−^ and O^−^) are generated exactly at the interface between the MnO_*x*_ and ceria lattice, the so-called Mn_*y*_Ce_1-*y*_O_*x*_ solid solution. Since the formation of a –Mn–O–Ce– bond would reduce the Coulomb interaction of Mn^δ+^–O^γ-^ or Ce^δ+^–O^γ-^, the formation energy of oxygen vacancies can be greatly lowered^34^. Several approaches to MnO_*x*_–CeO_2_ catalysts—such as the co-precipitation^31^,^32^, sol-gel^35^, combustion^33^, surfactant-assisted precipitation^36^ and hydrothermal methods^37^—have been developed. Unfortunately, traditional methods of preparing a MnO_*x*_–CeO_2_ catalyst often lead to the formation of multiphases with limited Mn_*y*_Ce_1−*y*_O_*x*_ solid solution, which is only observed at the interfaces between MnO_*x*_ and ceria nanocrystals. Recently, Yang and co-workers reported a general route to phase-pure transition-metal-substituted ceria nanocrystals via solution-based pyrolysis of bimetallic Schiff base complexes, but the ratio of transition metal substitution (10 mol %) is somewhat low^38^. Given that the solid solution phase of a MnO_x_–CeO_2_ catalyst is responsible for the low-temperature redox activity, a Mn_0.5_Ce_0.5_O_*x*_ solid solution with 50% manganese atoms doping into a ceria lattice may be an ideal candidate for catalytic oxidation, because maximum active oxygen species are expected in such a structure. In the view of synthetic chemistry, the biggest challenge for constructing a Mn_0.5_Ce_0.5_O_*x*_ solid solution with as high as 50% cerium atoms substituted by manganese atoms but retaining the cubic fluorite structure lies in controlling the homogenization with –Mn–O–Ce–bonds throughout the backbone (Fig. 1b).

In this contribution, we report an efficient, sustainable approach to a homogeneous Mn_0.5_Ce_0.5_O_*x*_ solid solution, whose ideal structure with Mn^4+^ ions in the ceria matrix is suggested by X-ray diffraction (XRD), X-ray photoelectron spectroscopy (XPS), scanning electron transmission microscopy–X-ray energy dispersive spectroscopy (STEM–XEDS) mapping analysis and H_2_ temperature-programmed reduction (H_2_-TPR). To the best of our knowledge, it is the first time for the ultrahigh concentration of Mn^4+^ ion to be stabilized in a ceria lattice^30^,^31^,^32^,^33^,^34^,^35^,^36^,^37^,^38^. The essence of the current strategy for fabricating a uniform Mn_0.5_Ce_0.5_O_*x*_ solid solution is the slow hydrolysis of Mn/Ce precursors at the surfaces of ionic liquid ‘supermolecular' networks. Surprisingly, a mesoporous structure with a high surface area is observed for the Mn_0.5_Ce_0.5_O_*x*_ solid solution after ionic liquids removal. This structure is highly advantageous in heterogeneous catalysis, since it can expose more surface oxygen species, and faster mass diffusion/transfer can be expected^39^. This versatile soft-templating method for well-defined mesopores can cover various oxide solid solutions even transition metal perovskites such as Co_0.5_Ce_0.5_O_*x*_, Cu_0.2_Mn_0.3_Ce_0.5_O_*x*_, and YMnO_3_. We show the outstanding activity of a Mn_0.5_Ce_0.5_O_*x*_ solid solution catalyst in the low temperature, heterogeneous oxidation of cyclohexane (100 °C, conversion: 17.7%, selectivity for KA oils: 81%) with molecular oxygen as the oxidant. It is significantly superior to the results of current technology (140–160 °C, conversion: 3–5%); this process could be extended to the selective oxidation of various allylic or benzyl C–H bonds with the corresponding alcohols/ketones as products. This study provides a simple general strategy to obtain a mesoporous Mn_0.5_Ce_0.5_O_*x*_ solid solution catalyst that can make selective, O_2_-based oxidation of *sp*^*3*^ C-H bonds at mild temperatures possible.

## Results

### Fabrication of Mn_0.5_Ce_0.5_O_*x*_ solid solutions

**Figure 1:**
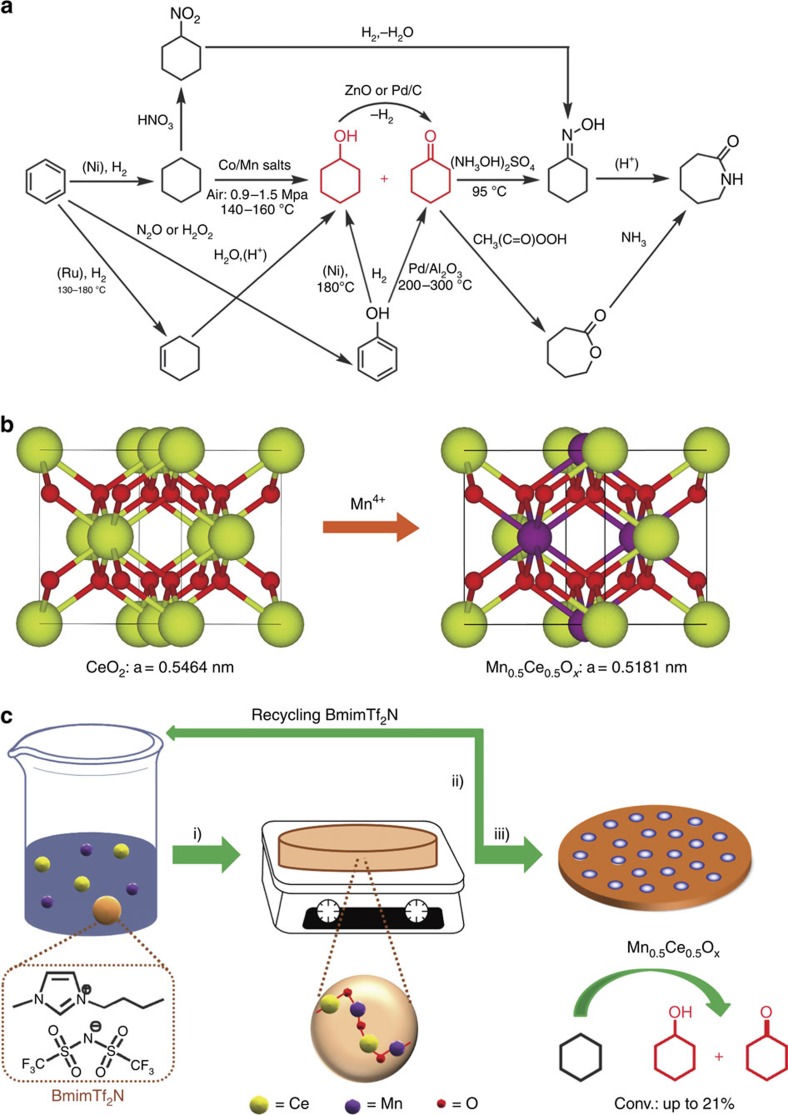
Synthetic and catalytic strategies. (**a**) A summary of state-of-art processes for caprolactam production (monomer for Nylon-6); (**b**) the evolution of doping 50% Mn^4+^ ions into a CeO_2_ lattice; (**c**) a solvent evaporation-induced self-assembly between metal salts and hydrophobic ionic liquid, reaction conditions: manganese (II) acetate, cerium (IV) methoxyethoxide and 1-butyl-3-methylimidazolium bis(trifluoromethanesulfonyl)imide (BmimTf_2_N) in ethanol: (i) stirring at room temperature for 2 h, and pouring into a petri dish at 50 °C for 24 h and 200 °C for 2 h, (ii) removing and recycling the BmimTf_2_N by Soxhlet extraction in ethanol (24 h), (iii) thermal treatment in air oven at 500 °C for 2 h.

The detailed route to the Mn_0.5_Ce_0.5_O_*x*_ catalyst is shown in Fig. 1c. In the present model system, manganese (II) acetate, cerium (IV) methoxyethoxide, 1-butyl-3-methylimidazolium bis(trifluoromethanesulfonyl)imide (BmimTf_2_N) and ethanol were mixed at a ratio of (1.0:1.8:1.6:8.0 w/w/w/w), and stirred at room temperature for 2 h. The dark red homogeneous solution was poured into a petri dish to evaporate solvents at 50 °C for 24 h, followed by solidification of the sample at 200 °C for 2 h with the formation of a primary metal oxo matrix around hydrophobic BmimTf_2_N via electrostatic interaction; and a frizzy solid film formed (Supplementary Fig. 1). It should be emphasized that the initial treatment temperature (200 °C) was higher than values used during surfactants or block copolymers-induced processes (∼95–120 °C)^40^. The good thermal stability of BmimTf_2_N (decomposition temperature: >350 °C, Supplementary Fig. 2) results in its high tolerable temperature, which allows a higher condensation degree of Mn/Ce precursors for strong backbones, and therefore affords the possibility of recycling BmimTf_2_N. In previous methods for forming mesoporous metal oxides, the surfactants or block copolymers used as soft templates usually cannot be removed and recycled before calcinations; otherwise, the porosity would collapse^39^,^40^. However, the organic templates cannot survive during high-temperature treatment (for example, 500 °C) and this sacrificial behaviour obstructs their industrial application. In contrast, the structure-directing BmimTf_2_N template can be easily extracted and recovered by refluxing in ethanol (Supplementary Figs 3–4), resulting in Mn_0.5_Ce_0.5_O_*x*_@200. The as-made sample was thermally treated at 500 °C for 2 h in air (Mn_0.5_Ce_0.5_O_*x*_@500).

### Characterization of mesoporous metal oxides

**Figure 2:**
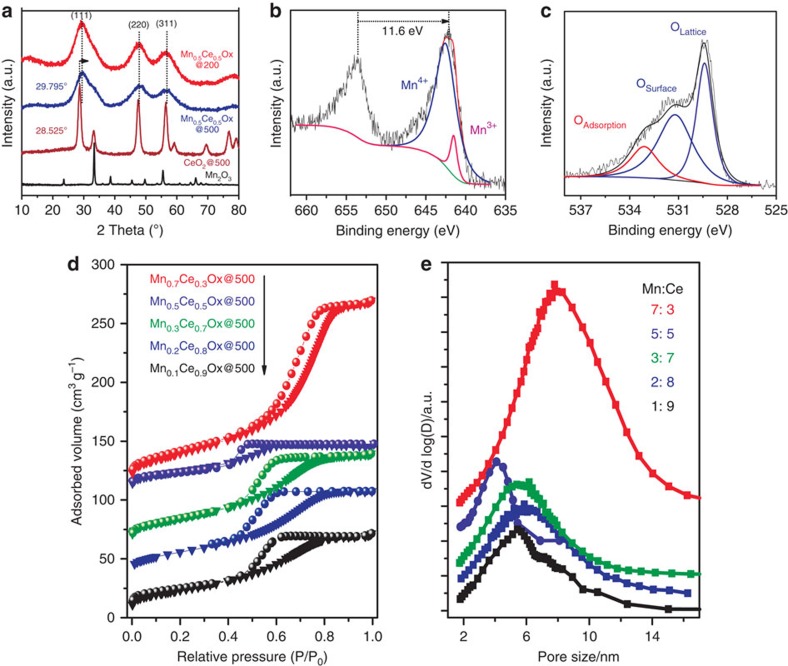
Structural characterizations of catalysts. (**a**) XRD patterns of Mn_0.5_Ce_0.5_O_*x*_@200, Mn_0.5_Ce_0.5_O_*x*_@500, CeO_2_@500 and Mn_2_O_3_. (**b**) XPS spectra of Mn 2p and (**c**) XPS spectra of O 1s of Mn_0.5_Ce_0.5_O_*x*_@500. (**d**) N_2_ sorption isotherm curves of Mn_*y*_Ce_1−*y*_O_*x*_@500 samples at 77 K; For clarity, the isotherm curves for Mn_0.2_Ce_0.8_O_*x*_@500, Mn_0.3_Ce_0.7_O_*x*_@500, Mn_0.5_Ce_0.5_O_*x*_@500 and Mn_0.7_Ce_0.3_O_*x*_@500 were offset by 30, 60, 105 and 105 cm^3^ g^−1^, respectively. (**e**) pore size distributions of Mn_*y*_Ce_1-*y*_O_*x*_@500 samples.

Figure 1b illustrates the evolution of crystal structures upon doping of 50% Mn^4+^ions into a ceria lattice, and a density functional theory calculation of structural models showed the change in the optimized lattice parameter *a*. Compared with ceria (*a*=0.5464, nm), an ideal Mn_0.5_Ce_0.5_O_*x*_ with a symmetrical Mn^4+^ substitution undergoes shrinkage along the *a* axis (*a*=0.5181, nm). This is reasonable because the ionic radii of manganese ions (Mn^4+^: 0.053 nm; Mn^3+^: 0.065 nm; Mn^2+^: 0.083 nm) are smaller in size than those of cerium ions (Ce^4+^: 0.097 nm; Ce^3+^: 0.114 nm). Indeed, the XRD pattern of the Mn_0.5_Ce_0.5_O_*x*_@500 sample showed a clear shift towards a higher Bragg angle compared with pure ceria and its corresponding lattice parameter *a* calculated by a (111) peak at 29.795° (*a*=0.5194, nm) was very close to the above theoretical result (*a*=0.5181, nm), revealing the possible replacement of Ce^4+^ by Mn^4+^ in the cubic fluorite structure (Fig. 2a, Supplementary Fig. 5, Supplementary Table 1). Extremely broad diffraction peaks for (111), (220) and (311) reflections of the Mn_0.5_Ce_0.5_O_*x*_@500 sample were observed. The average crystalline size was 1.4 nm, calculated by the Scherrer equation. The small crystalline size can be attributed to the confined hydrolysis and condensation of Mn/Ce precursors templated by the heterogeneous BmimTf_2_N structure^41^. In addition, a partially crystalline structure has already formed in the Mn_0.5_Ce_0.5_O_*x*_@200 sample.

To study the oxidation state of surface species, XPS spectra for the Mn 2p and O 1s core levels of the Mn_0.5_Ce_0.5_O_*x*_@500 sample were recorded and are shown in Fig. 2b,c. The XPS curve of Mn 2p exhibited two peaks at 653.7 and 642.1 eV, which can be attributed to the Mn 2p_1/2_ and Mn 2p_3/2_ states, respectively. The spin orbit splitting is Δ*E*=11.6 eV, close to the value of MnO_2_ (11.7 eV)^42^. In addition, the Mn 2p_3/2_ peak is fitted with a Shirley background and Gaussian-Lorenz model functions, and two peaks at 641.5 and 642.6 eV can be obtained, based on standard binding energy and previous literatures^32^,^42^. The observed binding energies suggest the co-existence of Mn^3+^ and Mn^4+^ ions, but Mn^4+^ species with 87% content dominate the surface, in accordance with the structural model discussed above. Meanwhile, the O 1s spectrum with a shoulder peak is very broad, possibly owing to the overlapping contributions of various oxygen species. The curve was then resolved with the model discussed above and fitted into three peaks. The peaks at 529.4, 531.2 and 533.1 eV are ascribed to lattice oxygen atoms (O^2−^, denoted as O_α_), surface oxygen species (for example, O_2_^−^, O_2_^2−^, O^−^, denoted as O_β_) and chemisorbed water and/or carbonates (denoted as O_γ_), respectively. It is well recognized in the literatures that the O_β_ species from defective sites with an unsaturated structure are of great importance in the catalytic oxidation process^32^,^37^. The surface atomic concentration was then calculated by integrating the peak areas of different oxygen species. The atomic ratio of "reactive" oxygen species (O_β_) can reach 44.1%, arguing for the great potential of this solid solution in catalytic oxidations.

The porous nature of Mn_0.5_Ce_0.5_O_*x*_ samples was evaluated by nitrogen sorption measurements at 77 K. The Mn_0.5_Ce_0.5_O_*x*_@200 sample was dominated by micropores with remarkable N_2_ uptake at low relative pressure and its specific surface area calculated by the Brunauer–Emmett–Teller (BET) method was 467 m^2^ g^−1^ (Supplementary Fig. 6). The rich porosity should be directed during the removal of BmimTf_2_N. It also can be concluded that the backbone of the Mn_0.5_Ce_0.5_O_*x*_ sample formed at 200 °C is strong enough to withstand the high pressure of molecular packing. Both XRD patterns and Fourier-transform infrared spectra of the Mn_0.5_Ce_0.5_O_*x*_@200 sample suggest that it is an oxide precursor with acetate anions incorporated in the matrix (Fig. 2a, Supplementary Fig. 7). A weak coordination-induced network containing Mn(OAc)_2_ and partially dehydrated cerium hydroxide were proposed for the Mn_0.5_Ce_0.5_O_*x*_@200 sample, wherein the close connection between manganese and cerium ions is the key to restructuring into a Mn_0.5_Ce_0.5_O_*x*_ solid solution during calcination. This confined restructuring can prevent the formation of separate bulk manganese or cerium oxide phases (Supplementary Fig. 8)^38^.

Thermal treatment of the Mn_0.5_Ce_0.5_O_*x*_@200 sample led to pore expansion, as shown by the pore size distributions of samples at different temperatures (200, 400, 500 and 600 °C); the pore expansion is possibly the result of the progressive growth of nanocrystals (Supplementary Fig. 9)^43^. It is Interesting that the Mn_0.5_Ce_0.5_O_*x*_@500 material possessed a characteristic type IV sorption isotherm with a H_1_ hysteresis loop, including a sharp capillary condensation step at *p/p*_0_=0.4–0.5. The pore diameter located in 3–6 nm with a narrow distribution, derived from the sorption branch of the isotherm by using Barrett–Joyner–Halenda model (Fig. 2d,e). The Mn_0.5_Ce_0.5_O_*x*_@500 was a typically mesoporous material with a BET surface area of 89 m^2^ g^−1^. A series of mixed oxide solutions with a different Mn/Ce atomic ratio (1:9, 2:8, 3:7, 7:3) were also prepared, and mesoporous structures with high surface areas were observed for those samples (Supplementary Table 2). The Mn_0.7_Ce_0.3_O_*x*_@500 sample possessed a specific surface area of 125 m^2^ g^−1^ with large mesopores around 10 nm. Moreover, the current solvent evaporation-induced assembly of binary Mn_0.5_Ce_0.5_O_*x*_ around the BmimTf_2_N template can easily be extended to more metal-oxide combinations with similar mesoporous structures, such as: Co_0.5_Ce_0.5_O_*x*_@500 (using another Period 4 transition metal: S_BET_=52 m^2^ g^−1^, pore size: ∼3 nm; Supplementary Figs 10–11), YMnO_3_@700 (transition metal perovskite: S_BET_=56 m^2^ g^−1^, pore size: ∼7.5 nm; Supplementary Fig. 12), and Cu_0.2_Mn_0.3_Ce_0.5_O_*x*_@500 (ternary metal oxide: S_BET_=78 m^2^ g^−1^, pore size: ∼4 nm; Supplementary Figs 13–14). In some cases, the pore size of the target material (for example, SiO_2_) can be precisely tailored on a mesoporous scale (for example, 3–40 nm), via adjusting the mass ratio between precursor molecules and BmimTf_2_N (Supplementary Fig. 15). Given that a large BmimTf_2_N aggregation is responsible for generating distances/pores between the primary oxide particles, polymerized BmimTf_2_N was then synthesized, which could lead to wider mesopores (Supplementary Figs 16–17).

**Figure 3:**
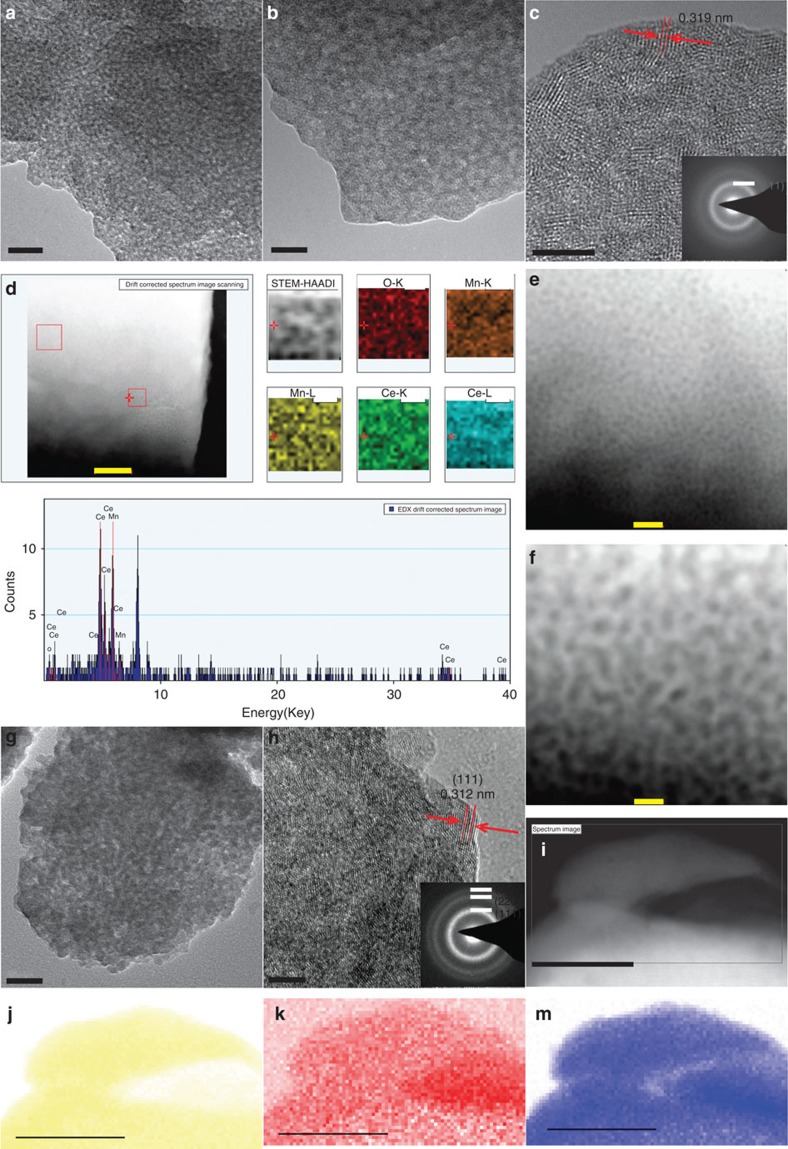
Studies of the catalyst by electron microscopy. (**a**–**c**) TEM/HRTEM images of Mn_0.5_Ce_0.5_O_*x*_@200 sample, the scale bar are 20, 10 and 5 nm, respectively. the inset in **c** is an electron microscopy pattern. (**d**) STEM-HAADF image of Mn_0.5_Ce_0.5_O_*x*_@200, scale bar, 100 nm; the corresponding XEDS of the O–K, Mn–K, Mn–L, Ce–K, Ce–L signals and XEDS. (**e**,**f**) STEM-HAADF image of Mn_0.5_Ce_0.5_O_*x*_@200 sample, scale bar, 20 nm and 10 nm, respectively. (**g**,**h**) TEM/HRTEM images of Mn_0.5_Ce_0.5_O_*x*_@500 sample, scale bar, 20 and 5 nm, the inset in **h** is an electron microscopy pattern. (**i**) STEM-HAADF image of Mn_0.5_Ce_0.5_O_*x*_@500 sample and the corresponding elemental mapping for Ce (**j**), Mn (**k**), O (**m**). Scale bar, 50 nm.

The transmission electron microscopy (TEM) and STEM in high-angular dark field mode (STEM-HAADF) images directly witness the evolution of Mn_0.5_Ce_0.5_O_*x*_ samples at different treatment temperatures. The Mn_0.5_Ce_0.5_O_*x*_@200 sample was rich in porosity with apparent pores of around 1–3 nm, in agreement with the value by nitrogen sorption measurement (Fig. 3a,b,e,f). Actually, the ionothermal synthesis of carbon materials (200 °C) in BmimTf_2_N solvent also resulted in a porosity within microporous domains. The clusters/aggregations of BmimTf_2_N, formed during interaction with the precursors, are more or less within 1–3 nm (ref. 44). This is understandable, since the density functional theory studies of ionic liquids suggest that imidazolium cations can form extended hydrogen bond interactions with up to three anions, leading to highly structured ionic liquid clusters of the minimal free energy^44^,^45^,^46^. High-resolution TEM (HRTEM) image of Mn_0.5_Ce_0.5_O_*x*_@200 showed some lattice fringes, and diffuse rings in the selected area electron diffraction patterns were observed (Fig. 3c). Therefore, the initial crystalline structure with a poor crystallinity has formed even at 200 °C. To verify the compositional details of Mn_0.5_Ce_0.5_O_*x*_@200, STEM–XEDS mapping analysis was carried out (Fig. 3d). In a 50 × 50 nm region, the Mn and Ce X-ray signals were evenly distributed and the atomic ratio of Mn:Ce was around 1:1 by energy-dispersive X-ray spectroscopy (EDS) with drift-corrected spectral imaging.

Thermal treatment at 500 °C can enlarge the pore size into the mesoporous range, as indicated by the TEM image of Mn_0.5_Ce_0.5_O_*x*_@500; the sample contains a high degree of the interstitial porosity between interconnected nanocrystals (Fig. 3g). It is worthy to note that no phases for separate MnO_*x*_ particles can be observed by HRTEM, further revealing the formation of a homogeneous solid solution. The HRTEM image of Mn_0.5_Ce_0.5_O_*x*_@500 exhibits clear lattice fringes and well-defined ring (in the electron diffraction pattern) structures of the (111), (220) and (311) planes for cubic ceria, implying extremely poor crystallinity and small crystal size, in accordance with the broad peaks in the XRD pattern (Fig. 3h). The homogeneous distribution of Mn and Ce atoms was also indicated by the STEM–XEDS mapping analysis (Fig. 3i–m).

**Figure 4:**
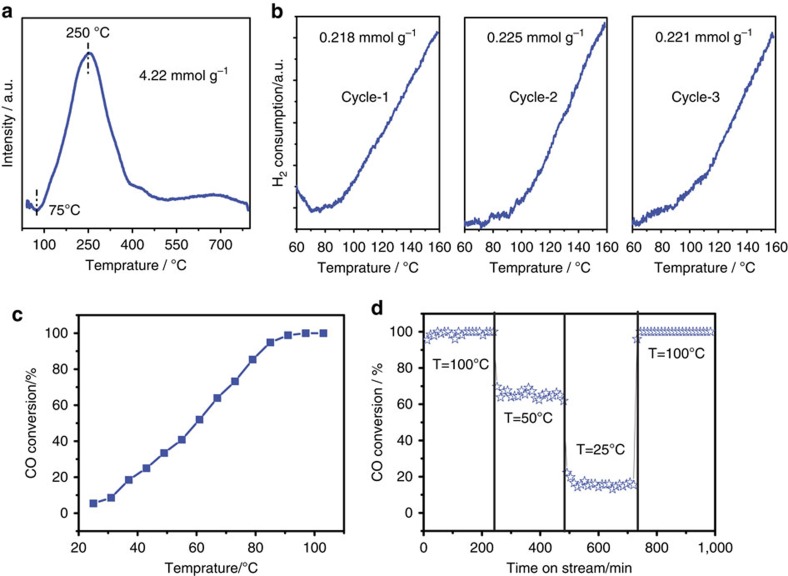
Redox property of the catalyst. (**a**) H_2_-TPR curve of Mn_0.5_Ce_0.5_O_*x*_@500 catalyst; (**b**) H_2_-TPR curve of Mn_0.5_Ce_0.5_O_*x*_@500 catalyst during H_2_ reduction-aerobic oxidation cycles; (**c**) Catalytic CO oxidation at different temperature over Mn_0.5_Ce_0.5_O_*x*_@500 catalyst; (**d**) Stability of Mn_0.5_Ce_0.5_O_*x*_@500 catalyst under CO oxidation and its catalytic performance during varied temperatures.

The H_2_-TPR profiles of the Mn_0.5_Ce_0.5_O_*x*_@500 sample are displayed in Fig. 4. Only one reduction peak was observed and located at ∼250 °C, a much lower temperature than the values from pure ceria (>500 °C) or MnO_*x*_ (350–600 °C; Fig. 4a)^31^,^32^,^33^. The reduction temperature of the Mn_0.5_Ce_0.5_O_*x*_@500 sample was also lower than those of the hybrid oxides with different Mn/Ce contents (Mn_0.1_Ce_0.9_O_*x*_@500, Mn_0.7_Ce_0.3_O_*x*_@500; Supplementary Fig. 18). This decreased reduction temperature can be attributed to the formation of a Mn_0.5_Ce_0.5_O_*x*_ solid solution with maximum –Mn–O–Ce– bonds, which can greatly lower the oxygen vacancy formation energy and enhance the mobility of oxygen species from the bulk to the surface to a large degree^34^. With CuO as the standard material, the H_2_ consumption of a Mn_0.5_Ce_0.5_O_*x*_@500 sample can reach 4.22 mmol g^−1^ and such a high value clearly suggests the large amount of the ‘active' oxygen species. If the chemical composition of our catalyst is assumed to be Mn^4+^Ce^4+^O_4–*x*_, the X value, based on the consumed H_2_, is calculated to be 1.1, in turn evidencing the doping of Mn^4+^ into the ceria lattice. Thus the H_2_-TPR peak can be assigned to the highly reducible manganese species with direct reduction from Mn^4+^ to Mn^2+^, along with partial surface Ce^4+^ reduction^47^. It is interesting that the reduction peak starts at 75 °C, in other words, that the oxygen vacancy is forming at such a low temperature, allowing the possibility of low-temperature catalysis. To probe the reversibility of active oxygen at low temperature, multiple redoxes of the Mn_0.5_Ce_0.5_O_*x*_@500 sample from 60 to 160 °C were carried out via a H_2_ reduction–aerobic oxidation cycle (Fig. 4b). During three cycles, the H_2_-TPR curves kept to the same trend and a similar amount of H_2_ consumption. By combining the unique properties of the current solid solution (for example, abundant active oxygen species, redox activity at low temperature and good stability) and the characteristic features of mesoporous materials (large pore size and high surface area), Mn_0.5_Ce_0.5_O_*x*_@500 contains most of the prerequisites for a noble metal-free heterogeneous catalyst to realize low-temperature selective oxidation of hydrocarbons by O_2_.

### Aerobic oxidation of cyclohexane by Mn_*y*_Ce_1−*y*_O_*x*_ Catalysts

Initial attempts to optimize the aerobic oxidation of cyclohexane were performed at 100 °C in the presence of different catalysts. A blank run without catalysts did not give any products in 4 h, suggesting that the auto-oxidation of cyclohexane by molecular oxygen cannot proceed under such a condition (Table 1, Entry 1). When catalysed by Mn_0.5_Ce_0.5_O_*x*_@500, the oxidation of cyclohexane occurred at 100 °C with a moderate conversion (6.5%) and a remarkable selectivity (95%) for KA oil (Table 1, Entry 2). As a noble metal-free solid catalyst, Mn_0.5_Ce_0.5_O_*x*_@500 indeed drives the aerobic oxidation of cyclohexane at a relatively low temperature. It should be emphasized that controlled oxidations of cyclohexane with CeO_2_@500, MnO_*x*_@500 or the physical mixture of CeO_2_@500 and MnO_*x*_@500 cannot proceed, confirming the synergistic action of manganese and cerium species in a Mn_0.5_Ce_0.5_O_*x*_@500 solid solution (Table 1, Entries 3–5). The Mn_0.5_Ce_0.5_O_*x*_@200 sample was also active for this process, which is reasonable since an initial crystalline structure has already formed at 200 °C (Table 1, Entry 6). The mixed oxides with various Mn/Ce atomic ratios (1:9, 2:8, 3:7 and 7:3) were also tested in the cyclohexane oxidation (Table 1, Entries 7–10). The optimal ratio was ∼1:1, in accordance with the H_2_-TPR results. The maximum –Mn–O–Ce– bonds throughout the matrix of the Mn_0.5_Ce_0.5_O_*x*_@500 solid solution may be responsible for its high activity, because more oxygen vacancies can be expected at low temperature.

The reaction temperature had a strong effect on the oxidation of cyclohexane. The cyclohexane conversion increased as the temperature increased from 80 to 150 °C; at the same time, a decreased selectivity for KA oil was observed (Table 1, Entries 11–13). It is interesting that oxidation can proceed at a temperature as low as 80 °C, which is in good agreement with the observation in H_2_-TPR that the active oxygen species is available above 75 °C. With the development of processes for low-temperature cyclohexane oxidation in mind, we focused on catalytic oxidation at 100 °C. The optimization of reaction time suggested that the reaction time of 12 h seemed to be a suitable time, and a 17.7% cyclohexane conversion with 81% selectivity for KA oil was obtained (Table 1, Entries 14–16). To probe the reaction pathway, two controlled runs were then performed. When the catalytic oxidation was carried out in argon, no detectable products were observed, giving evidence that molecular oxygen is the principal oxygen donor in the system (Table 1, Entry 17). In addition, the catalytic oxidation would be quenched, if hydroquinone, a free-radical scavenger, was added into the reaction system, which implied that the oxidation of cyclohexane may proceed through a radical chain mechanism (Table 1, Entry 18). The stability of the Mn_0.5_Ce_0.5_O_*x*_@500 catalyst was then investigated by cyclohexane oxidation for 4 h. After each run, the catalyst was recovered by centrifugation, and then carefully transferred into a reactor by the reaction solvent. The Mn_0.5_Ce_0.5_O_*x*_@500 worked well in at least 20 runs without significant activity loss, suggesting that the oxidation should run in a heterogeneous manner and it is a prerequisite for practical applications (Supplementary Figs 19–20). A possible reaction mechanism was then purposed, based on the results above, *in situ* diffuse reflectance infrared spectroscopy (DRIFTS) and *in situ* Raman spectra (Supplementary Fig. 21, Supplementary Note 1).

### Aerobic oxidation of hydrocarbons and CO

To probe the potential of this Mn_0.5_Ce_0.5_O_*x*_@500 solid solution, various hydrocarbons with *sp*^*3*^ C–H bonds were oxygenated at 110–120 °C (Table 2). Cyclohexene was oxidized with a moderate conversion to the mixture of 2-cyclohexen-1-one and 2-cyclohexen-1-ol (Table 2, Entry 1). The oxidation of ethylbenzene proceeded with high selectivity to acetophenone, although the ethylbenzene conversion was somewhat low (Table 2, Entry 2). Catalysed by Mn_0.5_Ce_0.5_O_*x*_@500 catalyst, the indane oxidation afforded a conversion of 75.4%, with 1-indanol and 1-indanone as the main products (Table 2, Entry 3). The catalyst also worked well in the oxidation of tetralin, a key step in the commercial production of α-naphthol (Table 2, Entry 4)^48^. Fluorene and diphenylmethane with a large molecular size could be transformed into fluorenone and diphenylmethanone, with high selectivity (Table 2, Entries 5–6). Therefore, it is probably fair to say that the Mn_0.5_Ce_0.5_O_*x*_@500 solid solution is a general catalyst for aerobic oxidation of allylic- or benzyl *sp*^*3*^ C–H bonds at relatively low temperature.

Actually, the same target for low temperature oxidation is also pursued in catalytic combustion, such as CO oxidation^32^,^36^,^38^. Encouraged by the interesting activity of Mn_0.5_Ce_0.5_O_*x*_@500 in O_2_ activation, we undertook a study of CO oxidation reaction over a Mn_0.5_Ce_0.5_O_*x*_@500 catalyst. The profile for CO conversion as a function of reaction temperature is presented in Fig. 4c. The Mn_0.5_Ce_0.5_O_*x*_@500 catalyst enables the 100% CO conversion at around 90 °C. It should be highlighted that the *T*_50_ (Temperature at which the 50% CO conversion is achieved) by Mn_0.5_Ce_0.5_O_*x*_@500 catalyst (60 °C) is lower than MnO_*x*_–CeO_2_ catalysts by other methods (co-precipitation method: 127 °C, surfactant-assisted method: 95 °C, hydrothermal method: 105 °C, citrate sol-gel method: 160 °C, Supplementary Table 3). The high activity of the Mn_0.5_Ce_0.5_O_*x*_@500 catalyst is attributed to the abundant superoxide species formed on the surface of the solid solution. The stability of Mn_0.5_Ce_0.5_O_*x*_@500 catalyst was also investigated, and it was found that 100% CO conversion at 100 °C can be preserved for 240 min (Fig. 4d). Moreover, the catalytic activity of the Mn_0.5_Ce_0.5_O_*x*_@500 sample was stable at different temperatures, and showed a rapid response to the temperature change. Notably, the Mn_0.5_Ce_0.5_O_*x*_@500 catalyst is active for CO oxidation even at room temperature (∼19% CO conversion).

## Discussion

In summary, we have shown the successful construction of mesoporous MnCeO_*x*_ solid solutions via a simple, effective and sustainable self-assembly strategy, which has at the same time been recognized in the fabrication of other hybrid metal oxides with well-defined mesopores. Experimental results reported herein, illustrate that the aerobic oxidation of cyclohexane to KA oil by Mn_0.5_Ce_0.5_O_*x*_@500 catalyst can proceed above 80 °C without any noble metal catalysts or sacrificial additives, and under optimized reaction conditions (100 °C), 17.7% cyclohexane conversion with 81% selectivity for KA oil was obtained. This finding could reinvigorate research into such a process for commercial exploitation, and thus make cyclohexane oxidation by a heterogeneous catalyst viable. In addition, selective oxidation of allylic or benzyl C–H bonds in various hydrocarbons were realized by the Mn_0.5_Ce_0.5_O_*x*_@500 catalyst using molecular oxygen as an oxidant. The versatility of Mn_0.5_Ce_0.5_O_*x*_@500 catalyst was also witnessed in CO oxidation with outstanding activity at a relatively low temperature (100% conversion at 90 °C).

Actually, the exceptional activity of the as-made catalyst can be the result of forming a Mn_0.5_Ce_0.5_O_*x*_ solid solution—which has been confirmed by a structural model, an XRD pattern, XPS analysis, TEM images, STEM–XEDX mapping analysis and an H_2_-TPR study—with several unique characteristics: (1) A high proportion (44.1%) of active oxygen species on the surface to promote O–O/C–H bond activation; (2) the introduction of 50 mol% Mn^4+^ ions into ceria matrix for the formation of maximum solid solution phases that can lower the energy for oxygen vacancy formation and benefit the rapid migration of oxygen vacancies from the bulk to the surface, thus continuing the activation of gas oxygen molecules; (3) a mesoporous structure for fast mass transfer/diffusion, and rich porosity to expose any more active sites ready for interaction with cyclohexane/O_2_. We expect that the Mn_0.5_Ce_0.5_O_*x*_ solid solution will provide a mild strategy for cyclohexane oxidation, and the manner of self-assembly with ionic liquids will inspire more designs of mesoporous oxide solid solutions for specific tasks in the near future.

## Methods

### Synthesis of Mn_0.5_Ce_0.5_O_*x*_ solid solution

In a typical synthesis of mesoporous Mn_0.5_Ce_0.5_O_*x*_ solid solution oxides, 6.16 g of cerium (IV) methoxyethoxide (18–20% in methoxyethoxide, Gesta), 0.63 g Mn(OOCCH_3_)_2_·6H_2_O (99%, Aldrich) and 1.0 g of ionic liquid (BmimTf_2_N) were dissolved in 5.0 ml of ethanol. The solution was stirred at room temperature for 2 h until Mn(OOCCH_3_)_2_·6H_2_O was completely dissolved. Subsequently, ethanol (5.0 ml) was added slowly with stirring. The mixed solution was gelled in an open petridish at 50 °C for 24 h and aged at 200 °C for 2 h, and a solid film was obtained. The ionic liquid was extracted by refluxing the sample with ethanol in a Soxhlet extractor for 24 h. The as-made sample (Mn_0.5_Ce_0.5_O_*x*_@200) was thermally treated at 500 °C for 2 h with the heating rate of 1 K min^−1^ in air, and the final sample denoted as Mn_0.5_Ce_0.5_O_*x*_@500. Other metal oxides were prepared by the same process except with different metal precursors. The materials were characterized by N_2_ adsorption (TriStar, Micromeritics) at 77 K, powder XRD (Panalytical Empyrean diffractometer with Cu Ka radiation *k*=1.5418 A° operating at 45 kV and 40 mA), thermogravimetric analysis (TGA 2950, TA Instruments), Fourier-transform infrared spectrum (PerkinElmer Frontier FTIR spectrometer) and H_2_-TPR (Auto chem II, Micromeritics).

### Typical procedure for the catalytic oxidation of CO

Catalytic CO oxidation was carried out in a fixed-bed reactor (U-type quartz tube) with inner diameter of 4 mm at atmospheric pressure. A 30 mg catalyst supported by quartz wool was loaded in the reactor. The feed gas of 1% CO balanced with dry air passed though the catalyst bed at a flow rate of 10 ml min^−1^, corresponding to a gas hourly space velocity of 20,000 ml (h gcat)^−1^.

### Typical procedure for the catalytic oxidation of cyclohexane

Catalytic oxidations of cyclohexane under pressured O_2_ were carried out in a Teflon-lined stainless steel batch reactor (PARR Instrument, USA). Typically, cyclohexane (10 mmol; calculated by weight), CH_3_CN (3 ml) and catalysts used as described in the manuscript were loaded into the reactor (total volume: 100 ml). The reactor was sealed, and then purged with O_2_ to replace the air for three times. The O_2_ pressure was increased to 1 MPa, and then the reactor was heated to the desired temperature in 15 min. Then, the reaction was carried out for the desired time with stirring (stirring rate: 1,500 r.p.m.). After reaction, the reactor was placed in ice water to quench the reaction, and the products were analysed by gas chromatography (GC) with internal standard (2-butanone). The structure of products and by-products was identified using Perkin Elmer GC–MS (Clarus 680-Clarus SQ 8C) spectrometer by comparing retention times and fragmentation patterns with authentic samples.

### Typical procedure for the catalytic oxidation of other hydrocarbons

In a typical oxidation, 1 mmol substrate, 1 mmol anisole (internal standard), 5 ml CH_3_CN and 30 mg Mn_0.5_Ce_0.5_O_*X*_@500 catalyst were added into a Teflon-lined stainless steel batch reactor. The reactor was sealed and purged with O_2_ to replace the air for three times. After increasing the O_2_ pressure to 1 MPa, the reactor was heated to the desired temperature in 20 min. Then, the reaction was carried out for the desired time with magnetic stirring (stirring rate: 1,500 r.p.m.). After the reaction, the reactor was placed in ice water to quench the reaction, and the products were analysed by GC and GC–MS.

### Method for *in situ* DRIFTS

*In situ* DRIFTS measurement was performed on a Nicolet Nexus 670 spectrometer equipped with a MCT detector cooled by liquid nitrogen and an *in situ* chamber (HC-900, Pike Technologies) which allows the sample heated up to 900 °C. The exiting stream was analysed by an online quadrupole mass spectrometer (OmniStar GSD-301 O_2_, Pfeffer Vacuum). Before measurement, the Mn_0.5_Ce_0.5_O_*x*_@500 powder (100 mg) was treated *in situ* at 500 °C in 20% O_2_/He (30 min) with a flow rate of 25 ml min^−1^ to eliminate water traces. After cooling to room temperature in a He flow (20 ml min^−1^), the background spectrum was collected for spectral correction, and background peaks were also collected at 100 and 150 °C, respectively. Then, cyclohexane stream (by bubbling with He 20 ml min^−1^) was introduced to the *in situ* chamber for adsorption and reaction.

### Method for raman spectroscopy

The procedure for Raman spectra collection: Raman spectra were excited with a 532 nm laser (LAS-NY532/50) and collected with Horiba JobinYvon HR800 (800mm optical length), with a diffraction grating of 600 grooves per mm, the scattered light was detected with a charge-coupled device, cooled to 203 K for thermal-noise reduction. The Raman spectra of samples were collected from 25 to 150 °C in the range of 100–4,000 cm^−1^ with two accumulations for each spectrum.

## Additional information

**How to cite this article:** Zhang, P. *et al.* Mesoporous MnCeO_*x*_ solid solutions for low temperature and selective oxidation of hydrocarbons. *Nat. Commun.* 6:8446 doi: 10.1038/ncomms9446 (2015).

## Supplementary Material

Supplementary Information Supplementary Figures 1-21, Supplementary Tables 1-3, Supplementary Note 1 and Supplementary References

## Figures and Tables

**Table 1:** Selective oxidation of cyclohexane under different conditions*.

**Entry**	**Catalyst**	***T*** **(°C)**	***t*** **(h)**	**Conv. (%)**	**Sel. (%)**	**K/A**†
1	Blank	100	4 h	<0.1%	—	—
2	Ce_0.5_Mn_0.5_O_*x*_@500	100	4 h	6.5%	95%	4.8
3	CeO_2_@500	100	4 h	<0.1%	—	—
4	MnO_*x*_@500	100	4 h	<0.1%	—	—
5	CeO_2_@500 (50 wt%) MnO_*x*_@500 (50 wt%)	100	4 h	<0.1%	—	—
6	Ce_0.5_Mn_0.5_O_*x*_@200	100	4 h	5.1%	92%	4.2
7	Ce_0.1_Mn_0.9_O_*x*_@500	100	4 h	0.4%	98%	7.5
8	Ce_0.2_Mn_0.8_O_*x*_@500	100	4 h	2.3%	96%	5.8
9	Ce_0.3_Mn_0.7_O_*x*_@500	100	4 h	2.6%	97%	6.1
10	Ce_0.7_Mn_0.3_O_*x*_@500	100	4 h	4.8%	98%	5.2
11	Ce_0.5_Mn_0.5_O_*x*_@500	80	4 h	1.0%	>99%	>99.0
12	Ce_0.5_Mn_0.5_O_*x*_@500	120	4 h	10.5%	84%	3.5
13	Ce_0.5_Mn_0.5_O_*x*_@500	150	4 h	18.8%	52%	5.4
14	Ce_0.5_Mn_0.5_O_*x*_@500	100	8 h	13.5%	90%	3.3
15	Ce_0.5_Mn_0.5_O_*x*_@500	100	12 h	17.7%	81%	3.6
16	Ce_0.5_Mn_0.5_O_*x*_@500	100	16 h	21.8%	63%	6.5
17‡	Ce_0.5_Mn_0.5_O_*x*_@500; in argon	100	4 h	<0.1%	—	—
18§	Ce_0.5_Mn_0.5_O_*x*_@500; hydroquinone 50 mg	100	4 h	<0.1%	—	—

Conv., conversion; Sel., selectivity.

^*^Reaction conditions: cyclohexane 10 mmol (842 mg), catalyst 30 mg, CH_3_CN 3 ml O_2_ 10 bar. Selectivity=[cyclohexanol+cyclohexanone]/[consumed cyclohexane] × 100; conversion=[consumed cyclohexane]/[initial cyclohexane] × 100, respectively.

^†^K/A=the molar ratio between cyclohexanone and cyclohexanol.

^‡^In Argon.

^§^With hydroquinone 50 mg as additive.

**Table 2:**
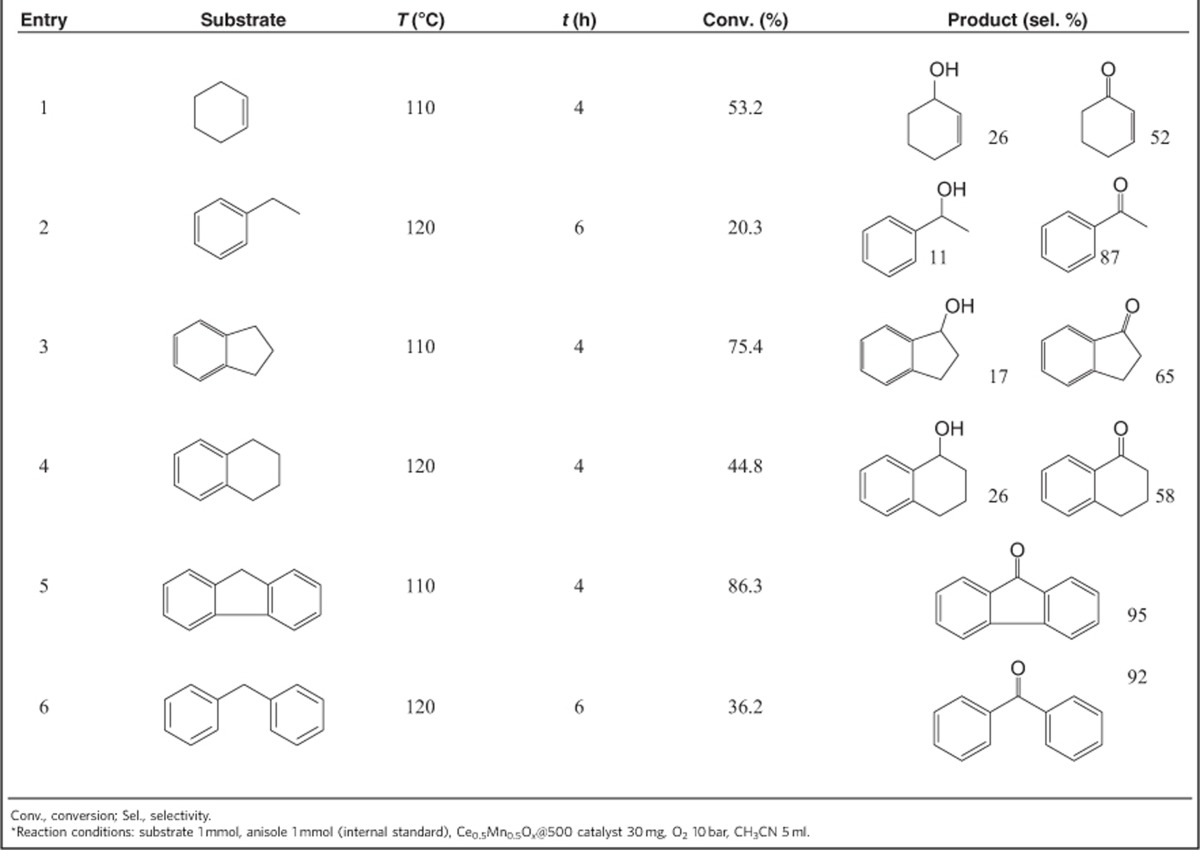
Selective oxidation of different hydrocarbons by a Mn/Ce catalyst^*^.
