# Supplementary material for: Mesoporous MnCeOx solid solutions for low temperature and selective oxidation of hydrocarbons
Source: Nat Commun. 2015 Oct 15;6:8446. doi: 10.1038/ncomms9446 (PMC4633985; doi:10.1038/ncomms9446)
Supplement: Supplementary Information — Supplementary Figures 1-21, Supplementary Tables 1-3, Supplementary Note 1 and Supplementary References [file ncomms9446-s1.pdf]

Supplementary Figure 1.

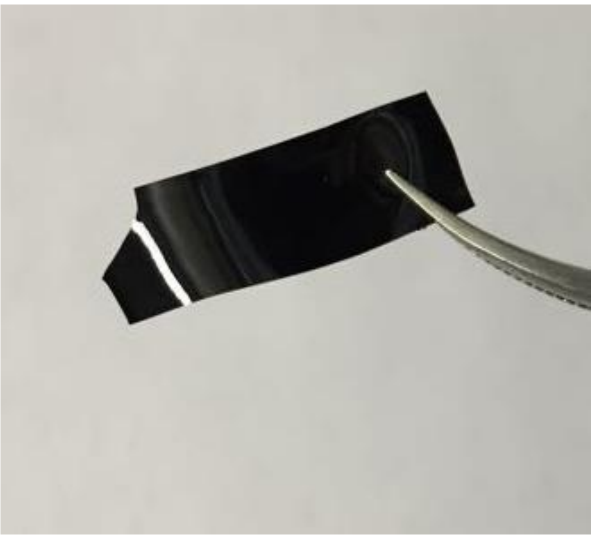

A film of primary $Mn_{0.5}Ce_{0.5}O_x$@200 sample with $BmimTf_2N$.

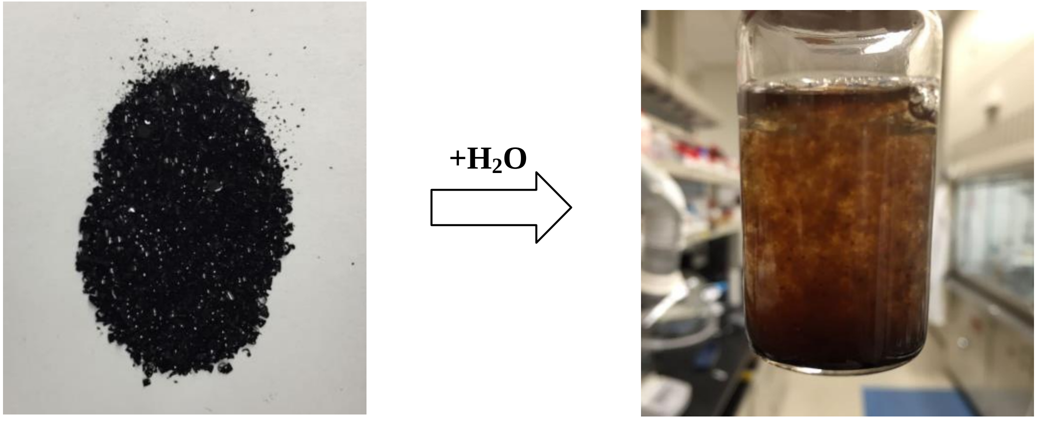

When the $Mn_{0.5}Ce_{0.5}O_x$@200 sample (after $BmimTf_2N$ removal) was added into water, a brown gel-like solid formed and well dispersed in water.

Supplementary Figure 2. TGA curve of $BmimTf_2N$ in N_2 atmosphere, heating rate: 10ºC min^{-1}.

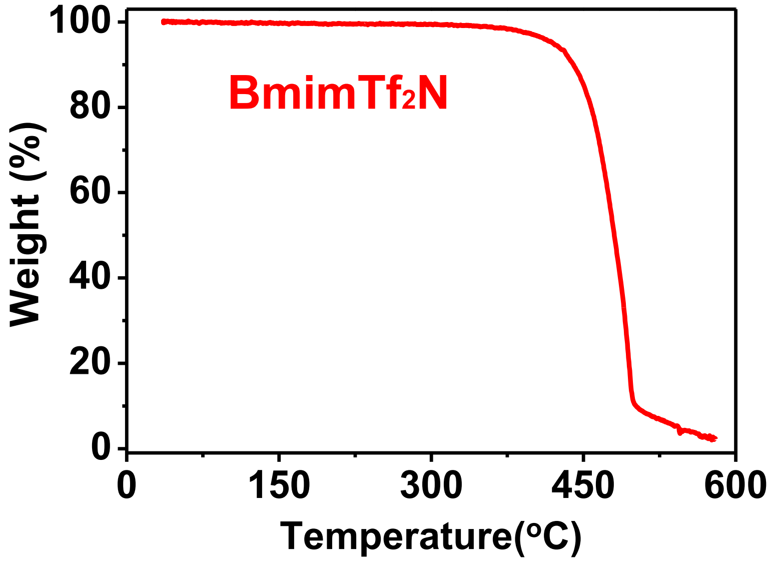

Supplementary Figure 3. FTIR curves of fresh and recycled $BmimTf_2N$. FTIR spectroscopy of recycled $BmimTf_2N$ showed those characteristic sorption bonds of fresh $BmimTf_2N$, suggesting the successful recycling of $BmimTf_2N$. Moreover, the recycled $BmimTf_2N$ is also colorless liquid, which is the same with fresh $BmimTf_2N$.

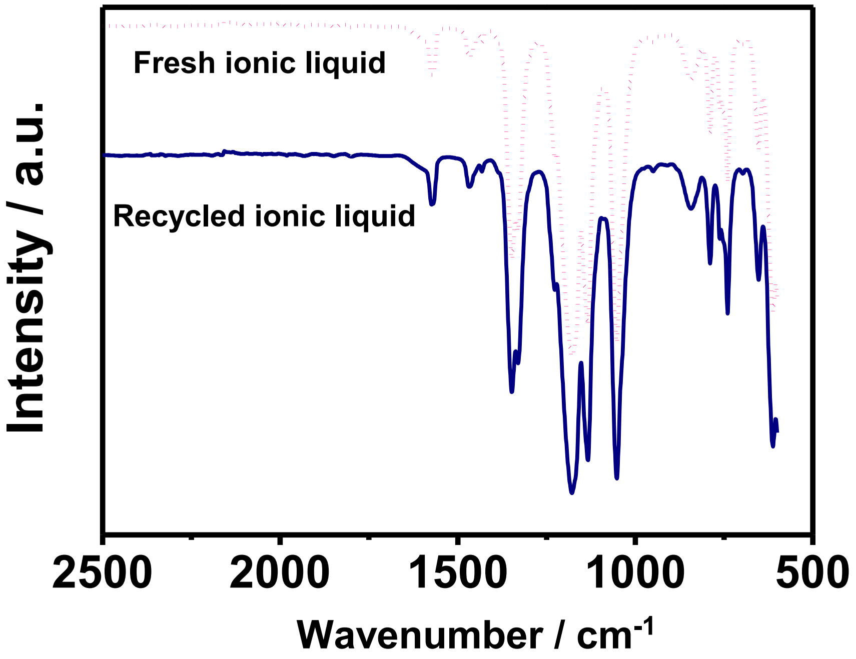

Supplementary Figure 4. FTIR curves of $Mn_{0.5}Ce_{0.5}O_x$@200 and $Mn_{0.5}Ce_{0.5}O_x$@200 with ionic liquids. After refluxing in ethanol, the BmimTf$_2$N incorporated in the pore of $Mn_{0.5}Ce_{0.5}O_x$@200 was removed, since the characteristic peaks of BmimTf$_2$N [ν_a(CF_3)1185 cm^{-1}, ν_s(SO_2)1134 cm^{-1}; *J. Mater. Chem. A* 2015, 3, 7229] disappeared in the FTIR curve.

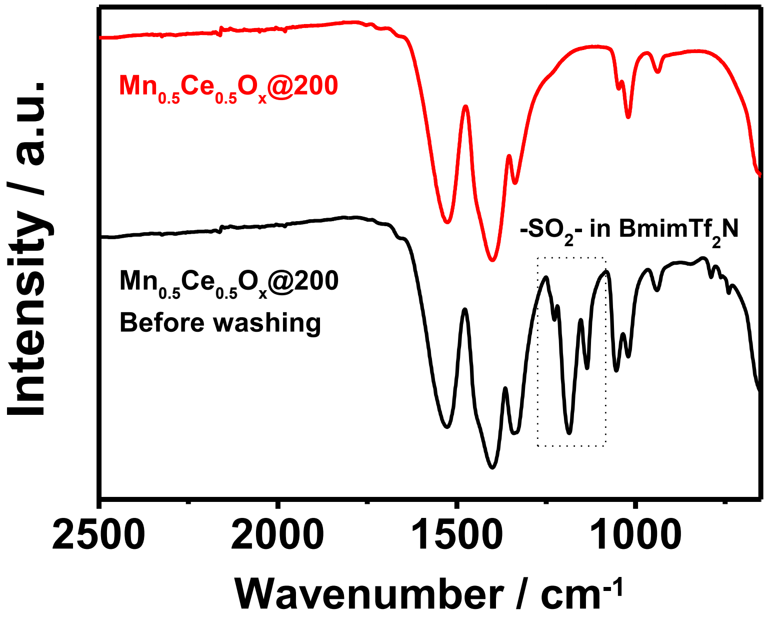

Supplementary Figure 5. A detailed analysis of XRD patterns of CeO_2, $Mn_{0.5}Ce_{0.5}O_x$@500-R ($Mn_{0.5}Ce_{0.5}O_x$@500-R was the reference sample prepared by citric acid-assisted method; *Chin J Catal, 2013, 34(2):25-33*) and $Mn_{0.5}Ce_{0.5}O_x$@500.

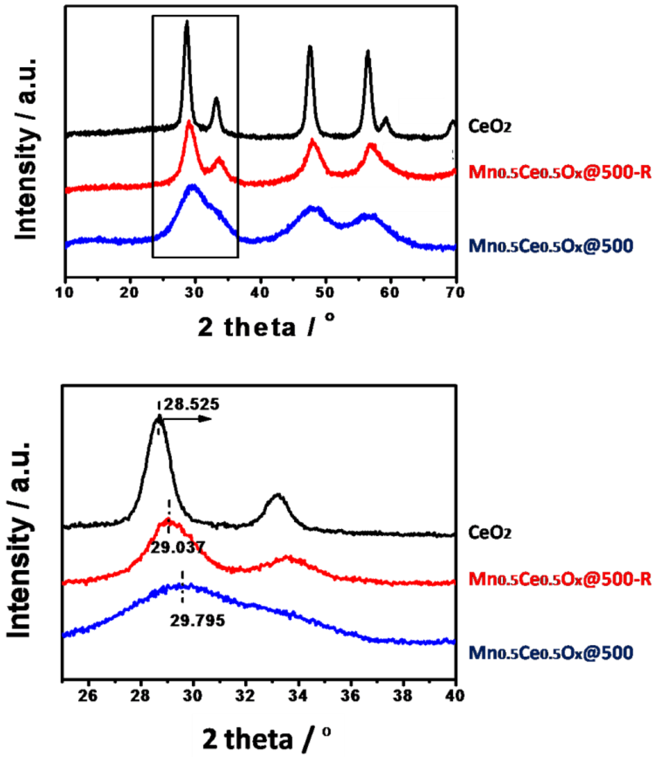

Supplementary Figure 6. N_2 sorption isotherm of $Mn_{0.5}Ce_{0.5}O_x$@200 sample at 77 K.

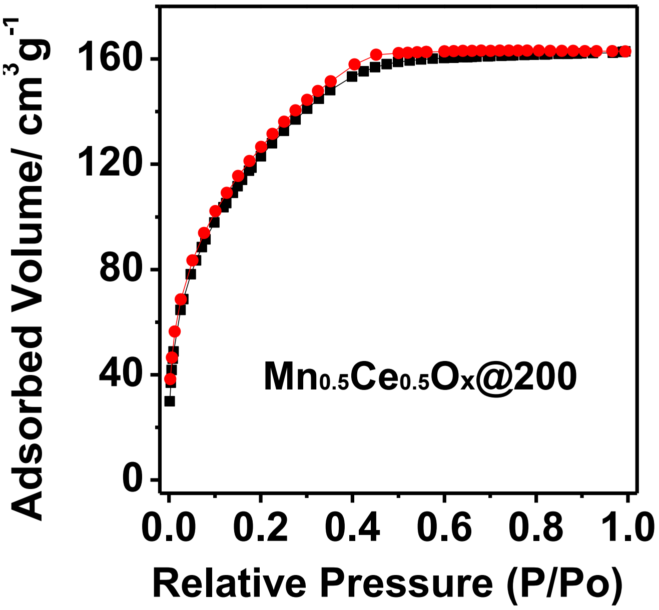

Supplementary Figure 7. FTIR of $Mn_{0.5}Ce_{0.5}O_x$ samples. The peaks at 1525 cm^{-1} and 1398 cm^{-1} of $Mn_{0.5}Ce_{0.5}O_x$@200 can be assigned to the vibration sorption of acetate ions, and no corresponding peaks were observed in $Mn_{0.5}Ce_{0.5}O_x$@500. As suggested by the characteristic sorption of acetate ions, it seems like that the $Mn(OAc)_2$ precursor was incorporated and preserved in the backbone of $Mn_{0.5}Ce_{0.5}O_x$@200, while the acetate ions would decompose under 500ºC treatment.

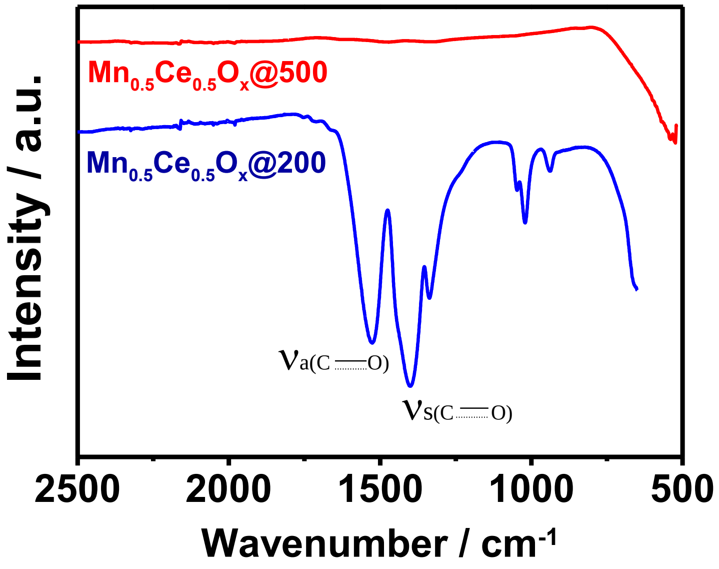

Supplementary Figure 8. A proposed chemical structure for $Mn_{0.5}Ce_{0.5}O_x$@200 sample with weakly coordinating interaction between Mn-Ce precursors.

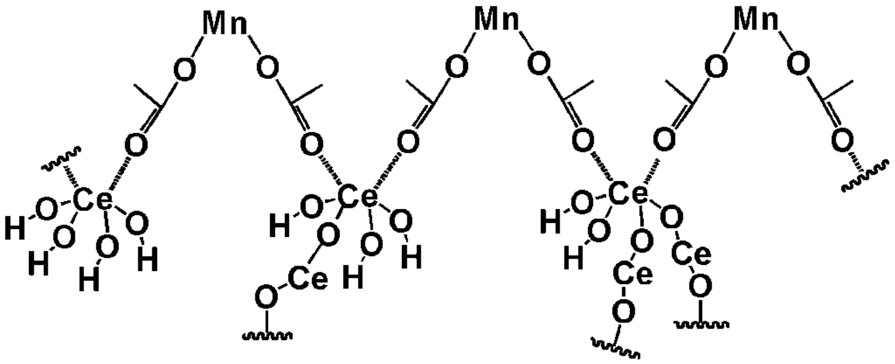

Mn0.5Ce0.5Ox@200

Supplementary Figure 9. Pore size distributions of $Mn_{0.5}Ce_{0.5}O_x$ samples under different treatment temperatures. They were obtained by analyzing N_2 sorption isotherms (77 K) with BJH methods. Under higher thermal treatment, the pore size of $Mn_{0.5}Ce_{0.5}O_x$ samples increased, such as: 200 ºC: <2 nm, 400 ºC: 2.4 nm, 500 ºC: 4.0 nm, 600 ºC: 15 nm. It is understandable if the re-structure and growth of Mn-Ce nanocrystals are considered, and it agrees well with the observation in the P123-mediated synthesis of MnO_x (*Nat. Commun*. **4**, 2952 (2013)).

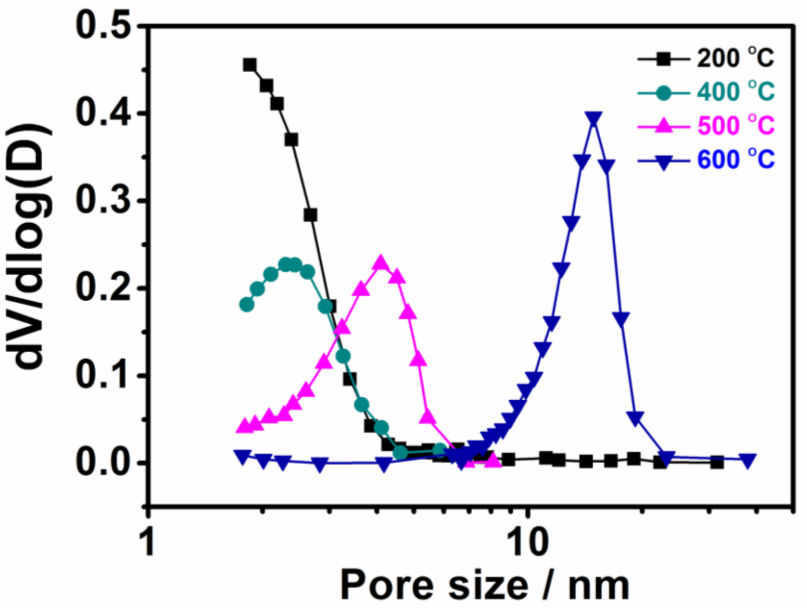

Supplementary Figure 10. N_2 sorption isotherm of $Co_{0.5}Ce_{0.5}O_x$@500 sample and the corresponding pore size distribution curve.

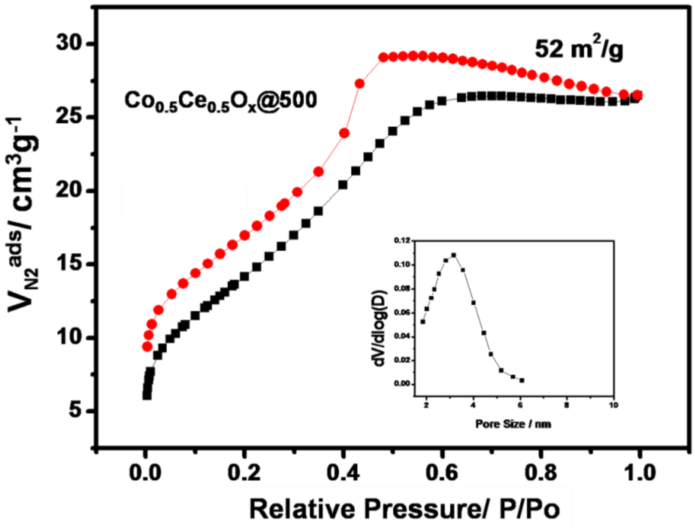

Supplementary Figure 11. XRD pattern of $Co_{0.5}Ce_{0.5}O_x$@500 sample. The curve suggests that the $Co_{0.5}Ce_{0.5}O_x$@500 sample was dominated by the cubic lattice of ceria.

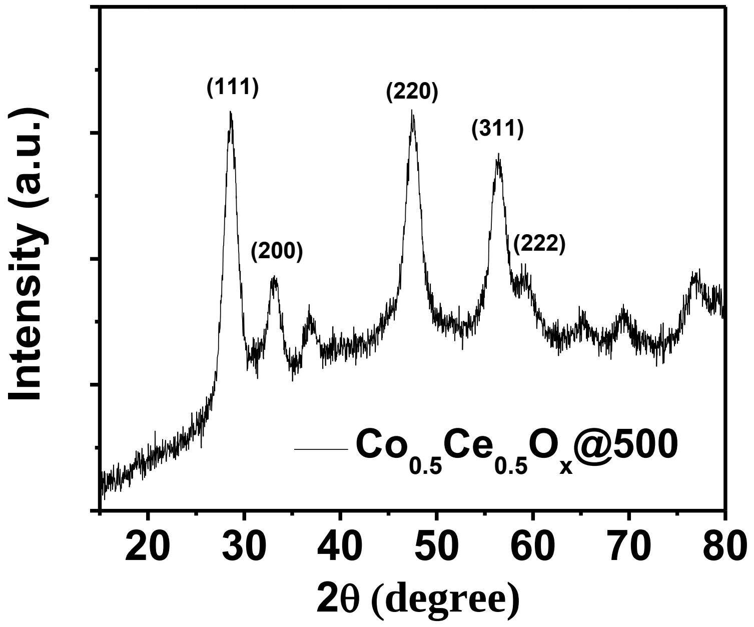

Supplementary Figure 12. N_2 sorption isotherm of $YMnO_3$ samples and the corresponding pore size distribution curves.

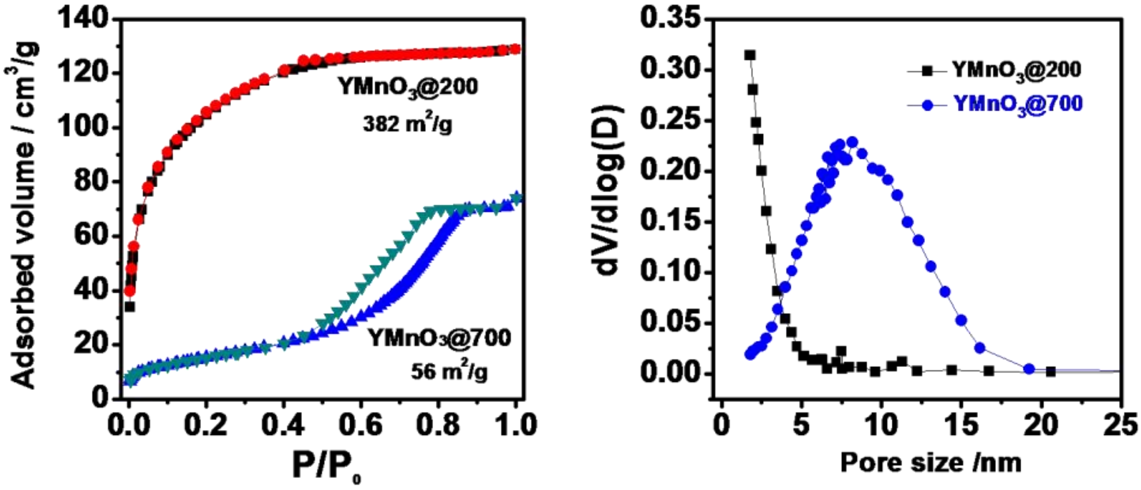

Supplementary Figure 13. N_2 sorption isotherm of $Cu_{0.2}Mn_{0.3}Ce_{0.5}O_x$@500 sample and the corresponding pore size distribution curve.

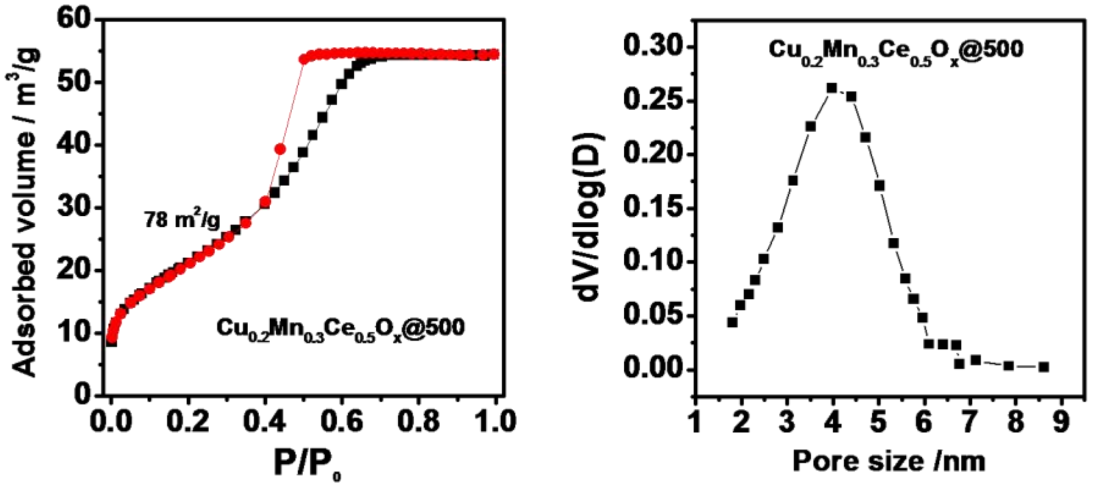

Supplementary Figure 14. XRD pattern of $Cu_{0.2}Mn_{0.3}Ce_{0.5}O_x$@500 sample. The $Cu_{0.2}Mn_{0.3}Ce_{0.5}O_x$@500 sample was made up of cubic fluorite structure of ceria, and no any separate phases for copper or manganese oxides were observed.

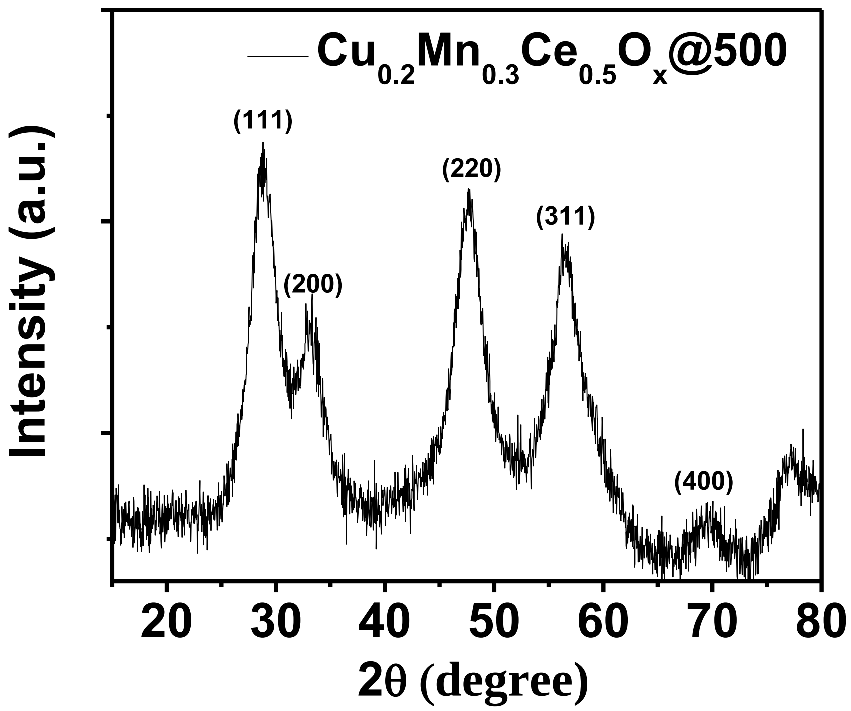

Supplementary Figure 15. a) N_2 sorption isotherm curves of SiO_2–x samples, while x represents the mass ratio between $BmimTf_2N$ and silica precursor (Tetraethylorthosilicate, TEOS). For clarity, the isotherm curves were offset by 300 cm^3/g for SiO_2–0.7, 800 cm^3/g for SiO_2–1.0, and 1400 cm^3/g for SiO_2–1.4. b) Pore size distribution curves. c) Relationship between pore size and x ($m_{BmimTf2N}/m_{TEOS}$). d) Relationship between surface area/pore volume of SiO_2 and x ($m_{BmimTf2N}/m_{TEOS}$). e) TEM image of SiO_2-0.35, f) TEM image of SiO_2-0.7, g) TEM image of SiO_2-1.4.

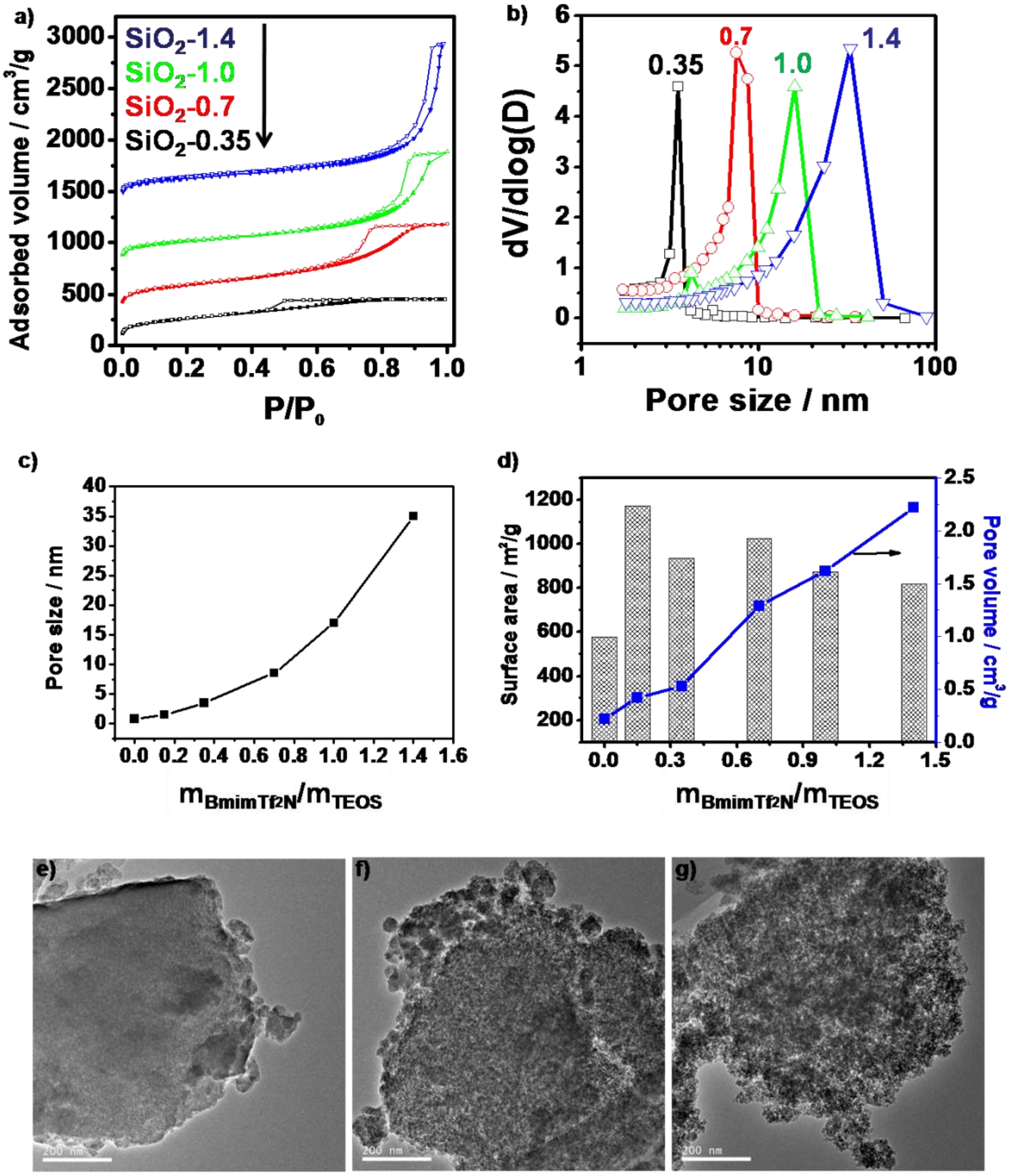

Supplementary Figure 16. a) The synthetic route to poly(BmimTf_2N), b) N_2 sorption isotherm of TiO_2-PIL@500 sample and c) the corresponding pore size distribution curve, d) N_2 sorption isotherm of TiO_2-IL@500 sample and e) the corresponding pore size distribution curve. Reaction conditions for TiO_2: $Ti(OC_4H_9)_4$ 1 mL, BmimTf_2N 1 g, ethanol 10 mL; or $Ti(OC_4H_9)_4$ 1 mL, Poly(BmimTf_2N) 0.1 g, ethanol 10 mL. Compared with IL-mediated synthesis of TiO_2 (100 wt%), significantly reduced amount (10 wt%) of Poly(BmimTf_2N) was needed for directing porosity. Both N_2 adsorption-desorption isotherms display the typical type IV curve with clear hysteresis loops, which are usually attributed to the predominance of mesopores. Interestingly, TiO_2-PIL@500 sample possesses wider pore size than TiO_2-IL@500, and it might be induced by the much bigger size of PIL.

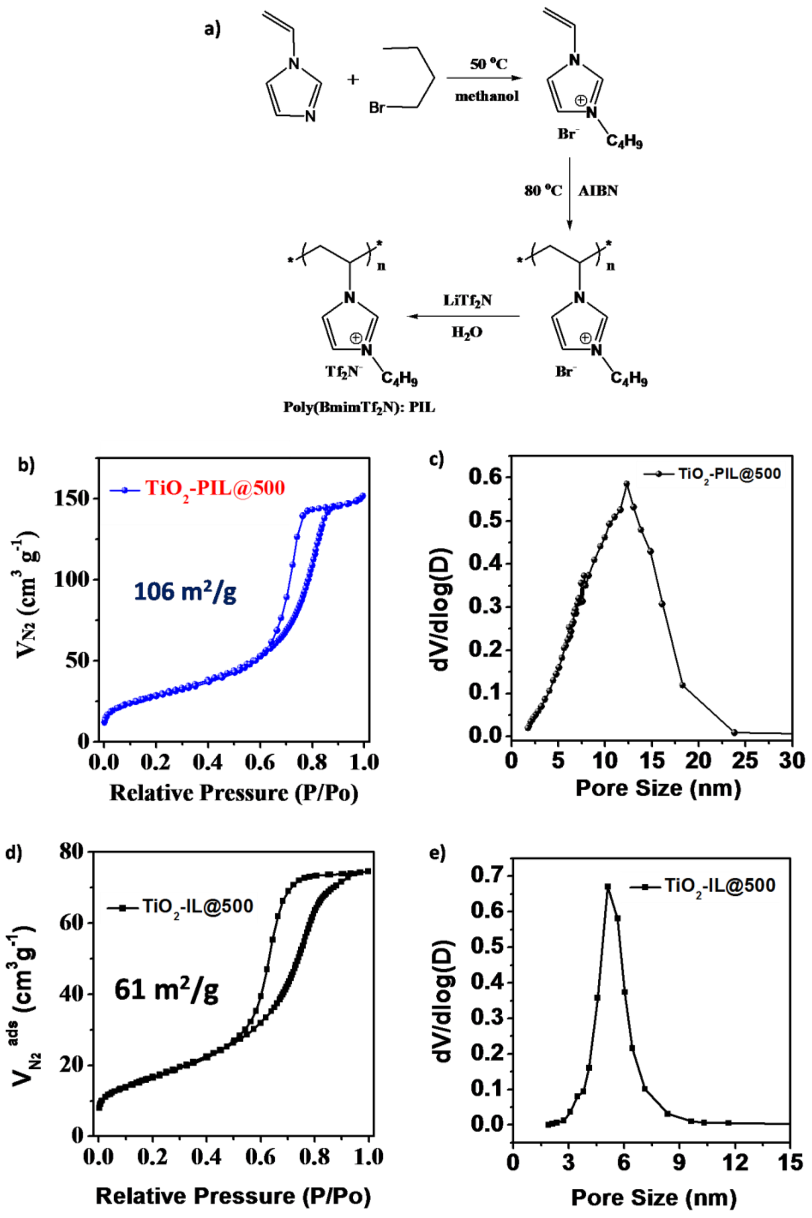

Supplementary Figure 17. N_2 sorption isotherm of SiO_2-PIL@500 sample and the corresponding pore size distribution curve. The PIL-mediated process was also be extended to the synthesis of mesoporous silica.

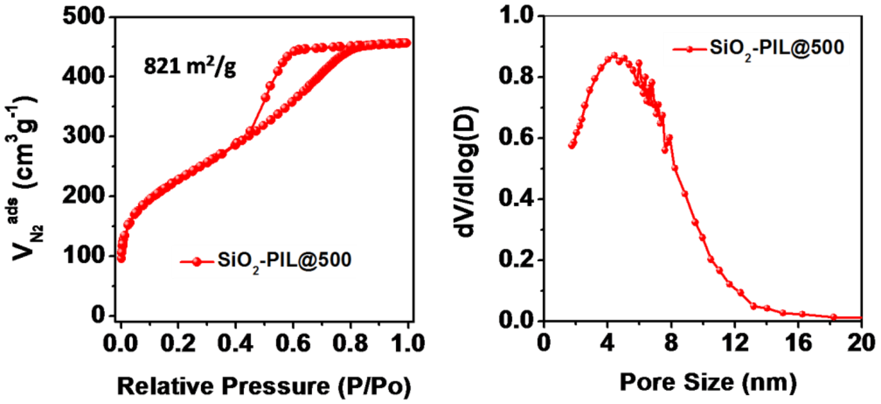

Supplementary Figure 18. H_2-TPR spectra of $Mn_{0.5}Ce_{0.5}O_x$@500, $Mn_{0.7}Ce_{0.3}O_x$@500 and $Mn_{0.1}Ce_{0.9}O_x$@500. H_2-TPR analysis is a powerful tool to understand the activity of surface oxygen specie. There is a clear difference between $Mn_{0.5}Ce_{0.5}O_x$@500 and $Mn_{0.1}Ce_{0.9}O_x$@500, and the latter displayed a much higher reduction temperature (250°C *vs*. 472°C). The $Mn_{0.7}Ce_{0.3}O_x$@500 sample with a high Mn content showed two reduction peaks at 328°C and 462°C, which is assigned to the reduction of MnO_2/Mn_2O_3 to Mn_3O_4, and the combined reduction of Mn_3O_4 to MnO and surface oxygen removal of ceria. The $Mn_{0.5}Ce_{0.5}O_x$@500 solid solution with more –Mn–O–Ce– bonds afforded better reducibility than other samples.

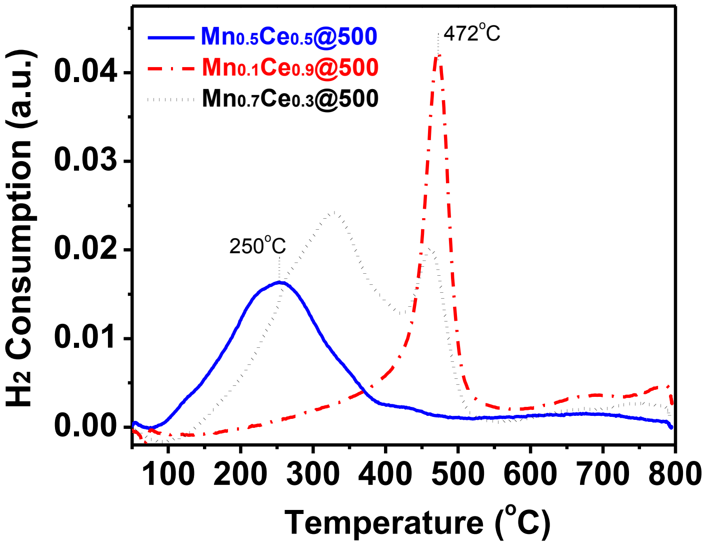

Supplementary Figure 19. Recycling runs for the aerobic oxidation of cyclohexane by $Mn_{0.5}Ce_{0.5}O_x$@500 catalyst; reaction conditions: cyclohexane 10 mmol, $Mn_{0.5}Ce_{0.5}O_x$@500 30 mg, $CHCN_3$ 3 mL, O_2 10 atm, 4 h. Conv.: conversion for cyclohexane, Sel.: selectivity for KA oil.

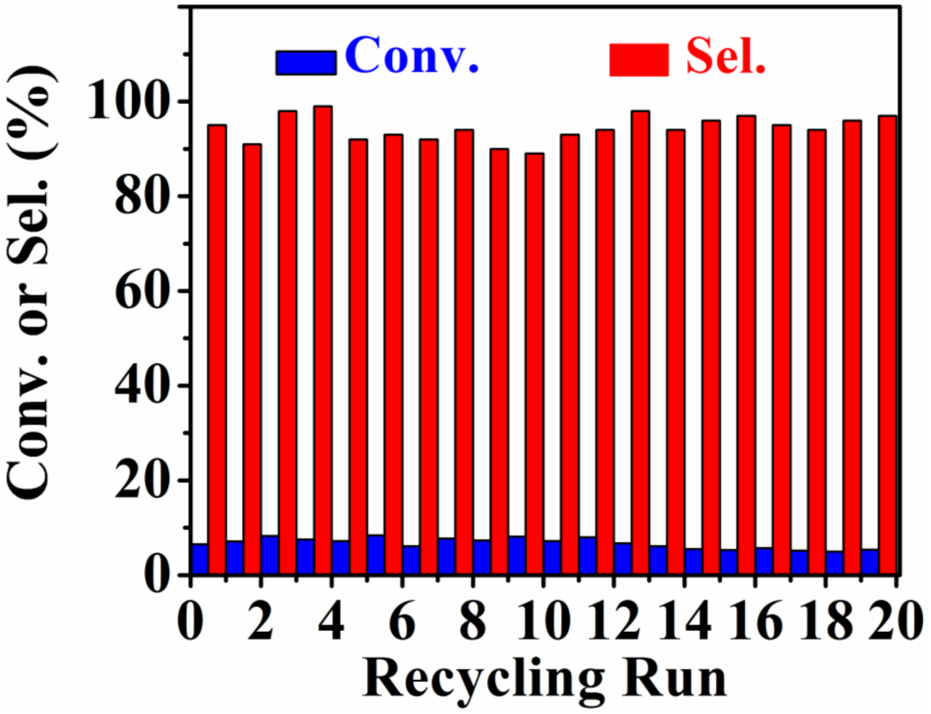

Supplementary Figure 20. The N_2 sorption isothermal of recycled $Mn_{0.5}Ce_{0.5}O_x$@500 catalyst. The BET specific surface area of $Mn_{0.5}Ce_{0.5}O_x$@500 catalyst is 70 $m^2\ g^{-1}$.

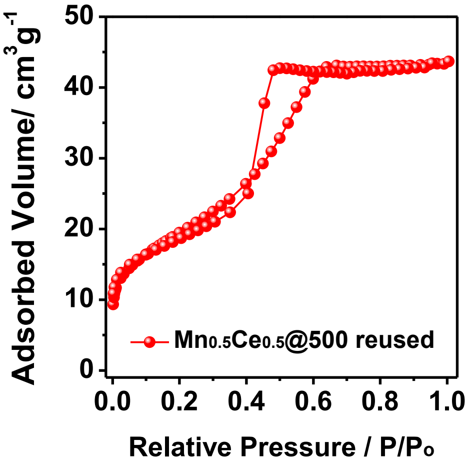

Supplementary Figure 21. In situ diffuse reflectance infrared spectroscopy of cyclohexane over the $Mn_{0.5}Ce_{0.5}O_x$@500 catalyst (Top left), In situ Raman spectra of cyclohexane/ $Mn_{0.5}Ce_{0.5}O_x$@500 in He (Top right), and a plausible reaction mechanism for the catalytic oxidation (Bottom).

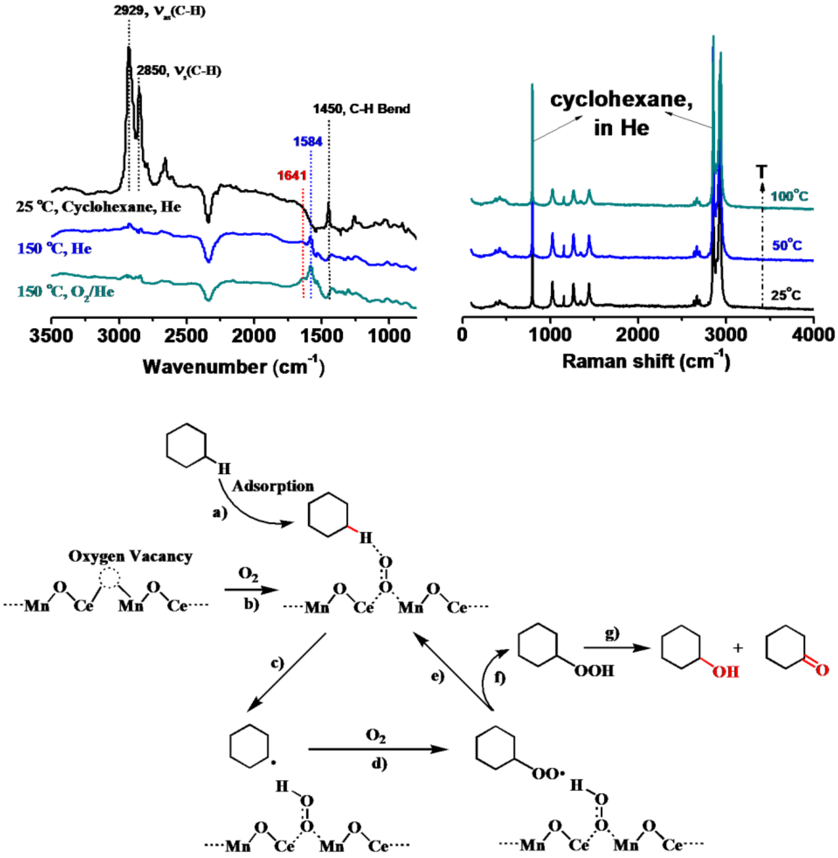

Supplementary Table 1. Characterization of as-made Mn-Ce samples.

Samples	2 theta (^{o})	Lattice constant *a* / nm	Crystallize size/ nm
$Mn_{0.5}Ce_{0.5}O_x$@500	29.795	0.5194	1.4
$Mn_{0.5}Ce_{0.5}O_x$@500-R	29.037	0.5327	4.0
CeO_2	28.525	0.5428	6.9

Supplementary Table 2. Pore structure parameters of Mn-Ce samples.

Samples	S_{BET} (m^2/g)	Pore volume (cm^3/g)
$Mn_{0.1}Ce_{0.9}O_x$@500	84	0.11
$Mn_{0.2}Ce_{0.8}O_x$@500	92	0.12
$Mn_{0.3}Ce_{0.7}O_x$@500	89	0.12
$Mn_{0.5}Ce_{0.5}O_x$@500	89	0.07
$Mn_{0.7}Ce_{0.3}O_x$@500	125	0.25

Supplementary Table 3. The activity of CeO_2-based oxides on CO oxidation.

Catalysts	Preparation method	GHSV/ ml/(g·h)	T_{50} and T_{90}	Ref
$Ce_{0.7}Mn_{0.3}O_2$	coprecipitation method, calcination at 500 °C in air	30,000	T_{50}=127°C T_{90}=170 °C	[1]
$Ce_{0.5}Mn_{0.5}O_2$	Hydrothermal method, calcination at 600 °C in N_2 and 400 °C in air	15,000	T_{50}=105 °C T_{90}=140 °C	[2]
$Ce_{0.6}Mn_{0.4}O_2$	Surfactant-assisted coprecipitation method, calcination at 500 °C in air	30,000	T_{50}=95 °C T_{90}=110 °C	[3]
$Ce_{0.9}Mn_{0.1}O_2$	Citrate sol-gel method, calcination at 600 °C in air	30,000	T_{50}=160 °C T_{90}=195 °C	[4]
**$Ce_{0.5}Mn_{0.5}O_x$**	**Ionic Liquid-mediated synthesis Calcination at 500 °C in air**	**20,000**	**T_{50}=60 °C T_{90}=85°C**	**This work**
$Ce_{0.9}Cu_{0.1}O_2$	Citrate sol-gel method, Calcination at 600 °C in air	30000	T_{50}=80°C, T_{90}= 100°C	[4]
$Ce_{0.9}Co_{0.1}O_2$	Citrate sol-gel method, Calcination at 600 °C in air	30000	T_{50}=150°C, T_{90}= 180°C	[4]
$Ce_{0.9}Ni_{0.1}O_2$	Citrate sol-gel method, Calcination at 600 °C in air	30000	T_{50}=130°C, T_{90}= 170°C	[4]
$Ce_{0.9}Fe_{0.1}O_2$	Citrate sol-gel method, Calcination at 600 °C in air	30000	T_{50}=190°C, T_{90}= 230°C	[4]
$Ce_{0.5}Co_{0.5}Cu_{0.1}O_2$	coprecipitation method. Calcination at 600 °C in air	48,000	T_{50}=57°C, T_{90}=70 °C	[5]
$Ce_{0.5}Zr_{0.5}O_2$	coprecipitation method, Calcination at 500 °C in air	30000	T_{50}=420°C, T_{90}= 527°C	[6]
$Ce_{0.8}Hf_{0.2}O_2$	coprecipitation method, Calcination at 500 °C in air	30000	T_{50}=300°C, T_{90}= 427°C	[6]
$Ce_{0.8}Tb_{0.2}O_2$	coprecipitation method, Calcination at 500 °C in air	30000	T_{50}=344°C, T_{90}= 407°C	[6]
$Ce_{0.8}Pr_{0.2}O_2$	coprecipitation method, Calcination at 500 °C in air	30000	T_{50}=383°C, T_{90}= 452°C	[6]

$Ce_{0.9}Fe_{0.1}O_2$	coprecipitation method<br>Calcination at 600 ^oC in air	30000	T_{50}=160^oC,<br>T_{90}= 230^oC	[7]

Supplementary Note 1.

To obtain more insight into the reaction process, we investigated the mechanism of the metal oxide–catalyzed oxidation of cyclohexane. **Supplementary Figure 21** illustrates a plausible reaction pathway. The adsorption of cyclohexane on the catalyst surface can be readily observed, as seen by *in situ* diffuse reflectance infrared spectroscopy (DRIFTS). After flowing the cyclohexane steam for 30 minutes, we changed to a pure helium atmosphere; the characteristic peaks of cyclohexane were preserved even with a helium purge at 150 ºC. The sorption of cyclohexane on the catalyst surface should proceed (Step a). As suggested by the XPS analysis of O 1s spectrum, active oxygen species are already rich at the interface. Interestingly, a new peak at 1584 cm^{-1} appeared when the *in situ* cell was heated to 150 ºC in helium. The hydrogen-abstraction from cyclohexane by active surface oxygen may occur with the formation of cyclohexane radicals (Step c). In the presence of 20%O_2/He, the peak at 1584 cm^{-1} increased in intensity, and a shoulder peak at 1641 cm^{-1} appeared, which could be assigned to the carbonyl group of cyclohexanone.[8] The Raman spectra of cyclohexane/$Mn_{0.5}Ce_{0.5}O_x$@500 in helium do not change from 25ºC to 100ºC, suggesting that molecular oxygen is acting as a terminal oxidant. It agrees well with the controlled oxidation (Table 1, Entry 17). This behavior becomes reasonable if the activation of gas molecular oxygen by surface oxygen vacancy for more active species is considered. Therefore, a channel, with a lower oxygen vacancy formation energy—rapid migration of oxygen vacancy for activating gas oxygen activation—abundant active surface oxygen for C-H activation, could be expected. The active cyclohexane radical might proceed the O_2-addition, hydrogen transfer process, and cyclohexyl hydroperoxide decomposition for the KA oil; [9-10] and the radical reaction mechanism has been suggested by our controlled reaction (Table 1, Entry 18).

Supplementary Reference

1. Venkataswamy, P.; Rao, K. N.; Jampaiah, D.; Reddy, B. M., Nanostructured manganese doped ceria solid solutions for CO oxidation at lower temperatures. Applied Catalysis B-Environmental 2015, 162, 122-132.
2. Zhan, W. C.; Zhang, X. Y.; Guo, Y. L.; Wang, L.; Guo, Y.; Lu, G. Z., Synthesis of mesoporous CeO2-MnOx binary oxides and their catalytic performances for CO oxidation. Journal of Rare Earths 2014, 32 (2), 146-152.
3. Zou, Z. Q.; Meng, M.; Zha, Y. Q., Surfactant-Assisted Synthesis, Characterizations, and Catalytic Oxidation Mechanisms of the Mesoporous MnOx-CeO2 and Pd/MnOx-CeO2 Catalysts Used for CO and C3H8 Oxidation. Journal of Physical Chemistry C 2010, 114 (1), 468-477.
4. Qiao, D. S.; Lu, G. Z.; Guo, Y.; Wang, Y. Q.; Guo, Y. L., Effect of water vapor on the CO and CH4 catalytic oxidation over CeO2-MOx (M=Cu, Mn, Fe, Co, and Ni) mixed oxide. Journal of Rare Earths 2010, 28 (5), 742-746.
5. Liu, Z. G.; Chai, S. H.; Binder, A.; Li, Y. Y.; Ji, L. T.; Dai, S., Influence of calcination temperature on the structure and catalytic performance of CuOx-CoOy-CeO2 ternary mixed oxide for CO oxidation. Applied Catalysis a-General 2013, 451, 282-288.
6. Reddy, B. M.; Thrimurthulu, G.; Katta, L., Design of Efficient Ce(x)M(1-x)O(2-delta) (M = Zr, Hf, Tb and Pr) Nanosized Model Solid Solutions for CO Oxidation. Catalysis Letters 2011, 141 (4), 572-581.
7. Qiao, D.; Lu, G.; Liu, X.; Guo, Y.; Wang, Y.; Guo, Y., Preparation of Ce(1-x) Fe (x) O(2) solid solution and its catalytic performance for oxidation of CH(4) and CO. Journal of Materials Science 2011, 46 (10), 3500-3506.
8. Corma, A.; Nemeth, L. T.; Renz, M. & Valencia, S. Sn-zeolite beta as a heterogeneous chemoselective catalyst for Baeyer-Villiger oxidations. Nature 2001, 412, 423-425.
9. Li, X. H.; Chen, J. S.; Wang, X. C.; Sun, J. H. & Antonietti, A. Metal-Free Activation of Dioxygen by Graphene/g-C3N4 Nanocomposites: Functional Dyads for Selective Oxidation of Saturated Hydrocarbons. *J. Am. Chem. Soc*. 2011, 133, 8074–8077.
10. Yu, H.; Peng, F.; Tan, J.; Hu, X.; Wang, H.; Yang, J. & Zheng, W. Selective Catalysis of the Aerobic Oxidation of Cyclohexane in the Liquid Phase by Carbon Nanotubes. Angew. Chem., Int. Ed. 2011, 50, 3978-3982.
